# Physiological Impact of a Synthetic Elastic Protein in Arterial Diseases Related to Alterations of Elastic Fibers: Effect on the Aorta of Elastin-Haploinsufficient Male and Female Mice

**DOI:** 10.3390/ijms232113464

**Published:** 2022-11-03

**Authors:** Quentin Boëté, Ming Lo, Kiao-Ling Liu, Guillaume Vial, Emeline Lemarié, Maxime Rougelot, Iris Steuckardt, Olfa Harki, Axel Couturier, Jonathan Gaucher, Sophie Bouyon, Alexandra Demory, Antoine Boutin-Paradis, Naima El Kholti, Aurore Berthier, Jean-Louis Pépin, Anne Briançon-Marjollet, Elise Lambert, Romain Debret, Gilles Faury

**Affiliations:** 1 Université Grenoble Alpes, Inserm, CHU Grenoble Alpes, HP2, U1300, 38000 Grenoble, France; 2 Institut de Biologie et Chimie des Protéines UMR5305-LBTI, CNRS, Lyon-7, Passage du Vercors, CEDEX 07, 69367 Lyon, France

**Keywords:** aorta, structure, mechanics, reactivity, elastic fiber synthesis/repair, pharmacotherapy, synthetic elastic protein, elastin haploinsufficiency

## Abstract

Elastic fibers, made of elastin (90%) and fibrillin-rich microfibrils (10%), are the key extracellular components, which endow the arteries with elasticity. The alteration of elastic fibers leads to cardiovascular dysfunctions, as observed in elastin haploinsufficiency in mice (*Eln^+/-^*) or humans (supravalvular aortic stenosis or Williams–Beuren syndrome). In *Eln^+/+^* and *Eln^+/-^* mice, we evaluated (arteriography, histology, qPCR, Western blots and cell cultures) the beneficial impact of treatment with a synthetic elastic protein (SEP), mimicking several domains of tropoelastin, the precursor of elastin, including hydrophobic elasticity-related domains and binding sites for elastin receptors. In the aorta or cultured aortic smooth muscle cells from these animals, SEP treatment induced a synthesis of elastin and fibrillin-1, a thickening of the aortic elastic lamellae, a decrease in wall stiffness and/or a strong trend toward a reduction in the elastic lamella disruptions in *Eln^+/-^* mice. SEP also modified collagen conformation and transcript expressions, enhanced the aorta constrictive response to phenylephrine in several animal groups, and, in female *Eln^+/-^* mice, it restored the normal vasodilatory response to acetylcholine. SEP should now be considered as a biomimetic molecule with an interesting potential for future treatments of elastin-deficient patients with altered arterial structure/function.

## 1. Introduction

The arteries all play the role of tubings, allowing for the circulation of blood from the heart to the organs. In a systemic circulation, arteries make it possible for blood to provide cells with oxygen, nutrients and different signaling molecules that they need [1]. This basic and vital role of systemic arteries is complemented by additional functions, which depend on their structure and location in the arterial tree. The large arteries located close to the heart, i.e., the aorta and the first part of its proximal branches, are responsible for the smoothing of the discontinuous flow and pulsatile pressure of the blood ejected by the heart (Windkessel effect) [2,3]. Smaller sized arteries and arterioles, located downstream of the arterial tree, participate less in the Windkessel effect and are more involved in the regulation of the distribution of the blood flow to the different organs, depending on the evolution of the need of each organ as a function of time or body activity [3,4].

The smoothing function of large arteries is made possible by their particular structure, with a large proportion of their wall being made of extracellular elastic fibers, which represent about 40–50% of the dry weight in the thoracic aorta [5,6]. The elastic fibers are arranged into multiple circumferentially oriented and concentric elastic lamellae, which provide the arteries with their main mechanical property—elasticity [7,8,9].

The main function of medium and small sized arteries, i.e., blood distribution to the organs, is related to the presence of a low number of elastic lamellae and a large proportion of vascular smooth muscle cells (VSMCs) in their walls, whose contraction reduces the vessel diameter and decreases blood flow. The differential contraction levels of VSMCs in the different arteries, made simultaneously possible by nervous or hormonal control, permit adjusting the perfusion of each organ to its exact need at any moment [1,4]. 

Systemic arteries could, however, undergo structural and functional alterations due to degradative aging processes, extrinsic deleterious events, such as sleep apnea/intermittent hypoxia, or genetic deficiency in some components of the elastic fibers made of ≈90% elastin (= polymer of the precursor tropoelastin), ≈10% microfibrillar components, especially fibrillin-1, and several quantitatively minor components, although of major importance, such as lysyl oxidase (LOX), which is responsible for the cross-linking of tropoelastin [10]. These conditions induce endothelial and/or smooth muscle cell dysfunctions as well as general remodeling of the extracellular matrix (ECM), including enzymatic or non-enzymatic degradation of elastic fibers. This results in abnormal reactivity and stiffening of the arteries, thereby limiting their action on blood pressure and flux smoothing, subsequently leading to altered hemodynamics and tissue perfusion [9,11,12,13].

Hemizygosity of the elastin gene in animals (*Eln^+/-^* mice) or humans, e.g., patients with Williams–Beuren syndrome (WBS) or isolated supravalvular aortic stenosis (SVAS), leads to narrower arteries, lower arterial elastin content, degraded, thinner and/or more numerous elastic lamellae, abnormal/stiffened elastic lamellae and large arteries, altered hemodynamics, cardiac hypertrophy and hypertension, as well as, in human patients, large artery stenoses [9,12,14,15,16,17,18,19]. Nevertheless, *Eln^+/-^* mice live relatively well, while Williams syndrome patients could present severe deterioration of their cardiovascular function, in particular due to the presence of aortic stenoses, drastically increasing the cardiac postcharge, limiting downstream perfusion and potentially leading to death [12,14,17]. 

Physiologically, in adults, the degradation of elastic fibers cannot be compensated for by their neosynthesis, since they are produced only until the end of childhood or adolescence, the elastic fiber stock/structures present at this step of life being progressively altered during adulthood and aging. Therefore, in order to prevent or treat the degradations of arterial elastic fibers and function occurring in the above-mentioned conditions, several pharmacological treatments aiming at limiting elastic fiber alterations or re-inducing elastic fiber formation by VSMCs have been evaluated [9]. In addition to some chemokines and growth factors [20], the intracellular concentration of Ca^2+^ ([Ca^2+^]i) also controls the synthesis of tropoelastin; increased [Ca^2+^]i stimulates ERK1/2 phosphorylation, which ultimately leads to decreased tropoelastin synthesis, and vice versa. [Ca^2+^]i can be modulated either directly by use of drugs regulating the calcium influx (calcium ionophores) or indirectly by molecules triggering cell membrane hyperpolarization (K^+^ channel openers), which closes the voltage-gated Ca^2+^ channel and stops Ca^2+^ influx [21,22,23]. Minoxidil, an ATP-dependent K^+^ (K_ATP_) channel opener and a potent antihypertensive vasodilator, has been shown to increase in vitro elastin synthesis by cultured VSMCs and in vivo arterial elastin content and/or elastic lamellae thickness in several (young or aged) rat and mouse models (spontaneously hypertensive rats (SHR), brown Norway (BN) rats, aged C57Bl/6J mice and *Eln^+/-^* mice) [21,24,25,26,27]. The chronic minoxidil treatment of mice, hemizygous for the elastin gene or not, also resulted in neosynthesis of the elastic fibers, protection of pre-existing elastic fibers, improvement of arterial elasticity and cerebral perfusion and lower blood pressure [25,27,28]. However, minoxidil treatment could induce undesirable side-effects, such as oedema or cardiac hypertrophy, reduced by the concomitant use of antidiuretics or other drugs, and presented a limited effect in a recent clinical trial [15]. Other K_ATP_ channel openers also stimulate elastin synthesis—in vivo for diazoxide or nicorandil [26,29], or in vitro in cultured rat VSMCs for pinacidil and some derivatives of cromakalim or diazoxide [26,30,31]. Chronic treatment of aged mice with dill extract also reactivated tropoelastin (*Eln*) and lysyl-oxidase-like-1 (*Loxl1*) gene expressions, protected pre-existing elastic lamellae and induced elastic fiber neosynthesis, and it decreased the aortic stiffness of the aging aortic wall, although the exact mechanisms are unknown [32].

These pharmacotherapeutic strategies were all based on the modulation of the cellular signaling pathways involved in arterial elastic fiber synthesis/protection and subsequent improvement of arterial mechanics in deleterious conditions, but they could present different undesirable side effects. Here, we evaluated the efficiency of a new strategy, aiming at directly improving the mechanical properties of the altered arterial elastic fibers by intravenous administration of an exogenous elastino-mimetic synthetic elastic protein (SEP, 55 kDa). SEP was designed to recapitulate the main physicochemical and biological functions of native tropoelastin (patent #WO2017194761). It includes highly hydrophobic domains with VGVA/LPG hexapeptide repeats, which also target the elastin receptor complex. These hydrophobic domains are interrupted by lysine-rich crosslinking sequences, and SEP terminates with the C-terminal domain 36 of human tropoelastin, which is known to favor its assembly onto microfibrils [33]. In vitro, SEP actively integrates elastin fibrillar deposits through LOX enzymatic activity (patent #WO2017194761). Here, we hypothesize that either a direct integration of SEP into the degraded aortic elastic fibers and/or a cellular signaling effect of SEP could improve the altered elastic properties of *Eln^+/-^* mice. An action of SEP on cellular signaling is expected, since it includes sequences recognized by integrin αvβ3 [34] and the elastin receptor complex (ERC), known to be involved in elastin metabolism and cell function [34,35,36,37] and present at least in endothelial and vascular smooth muscle cells [35,38]. In the present study, this strategy was tested in *Eln^+/-^* mice and was shown to be efficient in improving the arterial structure/function and general cardiovascular condition of these animals.

## 2. Results

The statistical analysis of the results was performed by using three- or four-way ANOVAs (variables: sex, genotype, treatment and, where appropriate, aorta intraluminal pressure). In order to clarify the presentation of the results, only the statistical facts in relation to the impact of SEP treatment are presented in the main text of the article, while the statistical facts in relation to the impact of genotype or sex are presented in Supplementary Materials Data S1.

### 2.1. Body and Heart Weights

SEP treatment did not change the body weight in all animal groups, while it reduced hematocrit in females, not males, independently of the genotype. Total heart and left ventricle weights to body weight ratios, similar in untreated male and female animals, were found to be higher in males than in females in SEP-treated mice, independently of the genotype (Table 1).

### 2.2. Blood Pressure

**Figure 1:**
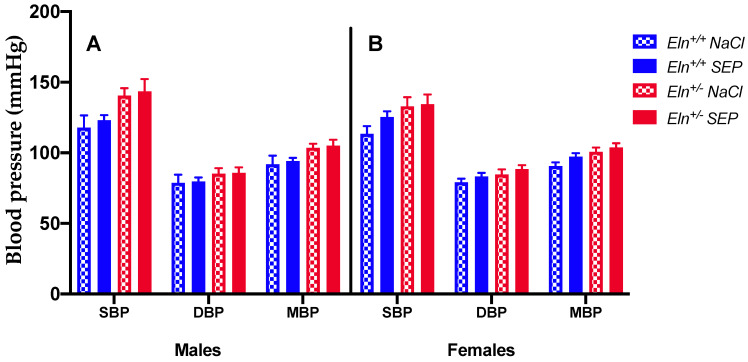
Systolic, diastolic and mean arterial blood pressures. The measurements were performed 3 days after NaCl (control) or SEP intravenous injection in the caudal vein of male and female mice of *Eln^+/+^* or *Eln^+/-^* genotypes. (**A**) Males, (**B**) Females, SBP: Systolic Blood Pressure, DBP: Diastolic Blood Pressure, MBP: Mean Blood Pressure. No significant effect of SEP treatment could be detected (four-way ANOVA and three-way ANOVAs for each sex, *p* > 0.05). Control animals were injected with the same volume of 0.9% NaCl. Values are mean ± SEM. n = 4–7 per group.

The treatment with SEP did not significantly change blood pressure in all animal groups (Figure 1).

### 2.3. Biomechanics of the Cannulated Ascending Aorta

**Figure 2:**
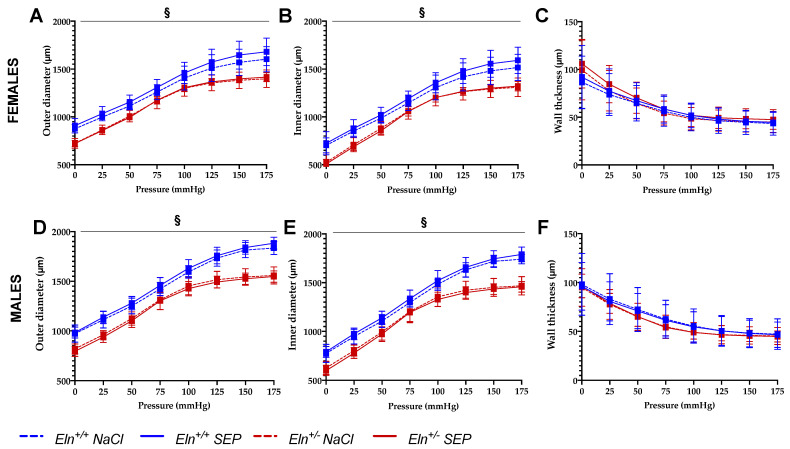
Diameter–pressure and wall-thickness–pressure curves of the cannulated ascending aorta. The measurements were performed by pressure arteriography 3 days after NaCl (control) or SEP intravenous injection in the caudal vein of male and female mice of *Eln^+/+^* or *Eln^+/-^* genotypes ((**A**–**C**): females; (**D**–**F**): males). (**A**,**D**): outer-diameter–pressure relation. (**B**,**E**): inner-diameter–pressure relation. (**C**,**F**): wall-thickness–pressure relation. ^§^ general significant interaction between genotype and treatment (three-way ANOVA, *p* ≤ 0.05), showing that SEP treatment induced a diameter elevation in *Eln^+/+^*, not *Eln^+/-^*, mice, independently of sex (post hoc Fisher’s least significant difference (LSD) tests, *p* ≤ 0.05). Control animals were injected with the same volume of 0.9% NaCl. Values are mean ± SEM. n = 7–10 per group.

Treatment with SEP induced an elevation of the aortic outer (OD) and inner (ID) diameters in *Eln^+/+^,* not *Eln^+/-^,* animals of both sexes (Figure 2A,B,D,E) and did not change the wall thickness in all groups (Figure 2C,F) compared to corresponding untreated mice.

**Figure 3:**
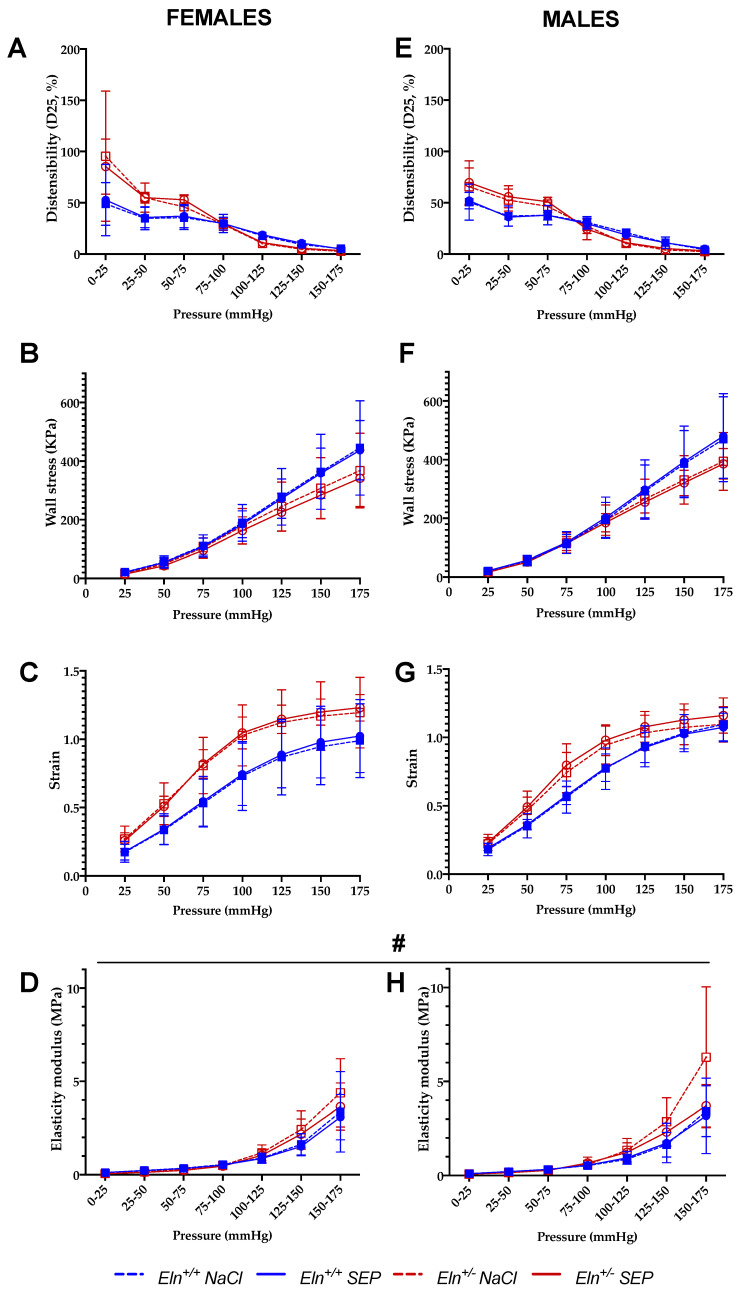
Mechanical parameters of the cannulated ascending aorta. The measurements were performed by pressure arteriography 3 days after NaCl (control) or SEP intravenous injection in the caudal vein of male and female mice of *Eln^+/+^* or *Eln^+/-^* genotypes. (**A**,**E**) Aortic distensibility (D25)–pressure increment relation; (**B**,**F**) circumferential stress–pressure relation; (**C**,**G**) circumferential strain–pressure relation; (**D**,**H**) incremental elastic modulus (Einc)–pressure increment relation. ^#^ general significant effect of treatment (NaCl control vs. SEP), independently of genotype, sex and pressure (four-way ANOVA, *p* ≤ 0.05). Control animals were injected with the same volume of 0.9% NaCl. Values are mean ± SEM. n = 7–10 per group.

No significant improvements of the aortic distensibility (Figure 3A,E), circumferential wall stress (Figure 3B,F) and wall strain (Figure 3C,G) were observed in SEP-treated mice compared to their untreated counterparts. Interestingly, the aorta incremental elastic modulus (Einc) (Figure 3D,H) was generally and significantly decreased in SEP-treated animals compared to untreated mice, independently of intraluminal pressure, genotype and sex (four-way ANOVA, *p* ≤ 0.05). The SEP-induced decrease in Einc was especially marked at 175 mmHg, independently of sex and genotype (four-way ANOVA followed by LSD test, *p* ≤ 0.05), and of greater amplitude in male mice (three-way ANOVA, *p* ≤ 0.05) (Figure 3D,H).

### 2.4. Ascending Aorta Ring Reactivity and Mechanics

**Figure 4:**
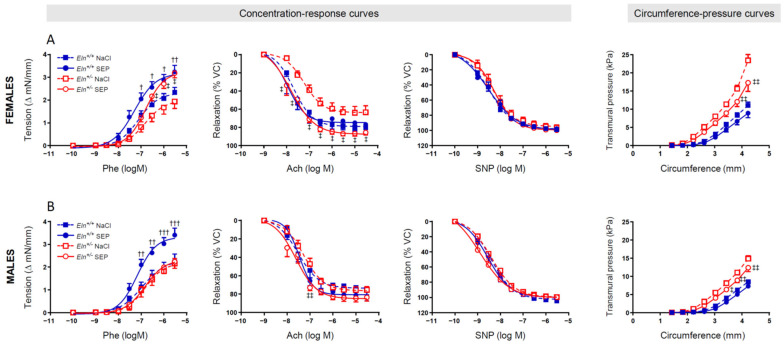
Concentration–response and circumference–pressure curves of the ascending aorta. The studies were performed by wire myography in aortae from female (**A**) and male (**B**) *Eln^+/+^* and *Eln^+/-^* mice treated with a single 0.9% NaCl (controls) or synthetic elastic protein (SEP) intravenous injection three days before the measurements. ^†^ *p* < 0.05, ^††^ *p* < 0.01 and ^†††^ *p* < 0.001: significant differences between *Eln^+/+^*SEP and *Eln^+/+^*NaCl; ^‡^ *p* < 0.05 and ^‡‡^ *p* < 0.01: significant differences between *Eln^+/-^*SEP and *Eln^+/-^*NaCl (two-way ANOVAs, followed, where necessary, by multiple Bonferroni post-tests). Values are mean ± SEM. n = 11–14 in each NaCl group; n = 7–8 in each SEP group.

The tension arteriography showed that, while mouse treatment with SEP significantly enhanced the phenylephrine (Phe)-induced vasoconstriction in *Eln^+/+^* mice of both sexes (at and above the Phe concentration 10^−7^ M), this effect in *Eln^+/-^* mice was present in females only, at and above the Phe concentration 3 × 10^−7^ M. Interestingly, SEP treatment also restored the blunted acute acetylcholine (Ach)-induced relaxation observed in female *Eln^+/-^* mice in a wide range of concentrations, starting from 10^−8^ M Ach, whereas it significantly increased the Ach-dependent vasodilatory response in male *Eln^+/-^* mice at the Ach concentration 10^−7^ M only. No intergroup difference was observed regarding the effect of acute sodium nitroprusside (SNP)-induced relaxation (Figure 4, three left panels).

The ascending aorta circumference–transmural pressure relation, indicative of arterial stiffness, was then studied. The SEP treatment did not significantly affect the circumference–pressure curves in *Eln^+/+^* mice, whereas it significantly reduced the transmural pressures in both female and male *Eln^+/-^* mice as the aortic rings were stretched to greater internal circumferences. The difference between *Eln^+/-^* and *Eln^+/+^* untreated mice persisted in animals treated with SEP (Figure 4, right panel).

### 2.5. Aorta Morphology and Structure

**Figure 5:**
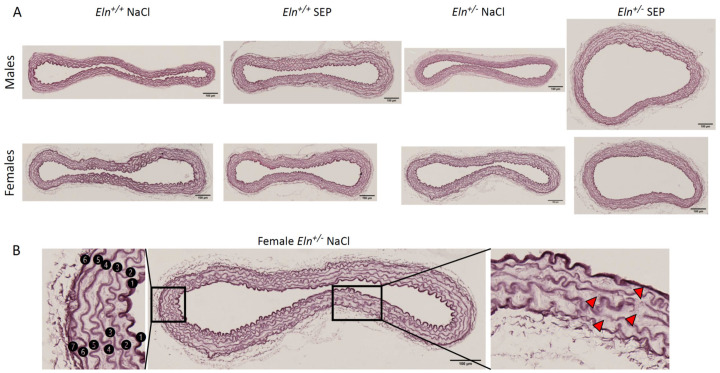
Elastic lamella detection by orcein staining in cross-sections of the distal part of the aortic arch of *Eln^+/+^* or *Eln^+/-^* male or female mice injected with SEP or 0.9% NaCl (control). (**A**) Representative images for each group. (**B**) Descriptive example, for an *Eln^+/-^* female mouse injected with 0.9% NaCl (control), of the process used for the quantification of elastic lamella abnormalities: (i) on the left, the numbering of each elastic lamella from the luminal side (#1: Internal elastic lamina) to the external side of the media (#6 or #7, depending on the vessel), and (ii) on the right, the identification of elastic lamella disruptions (red arrowheads). Scale bars = 100 µm.

**Figure 6:**
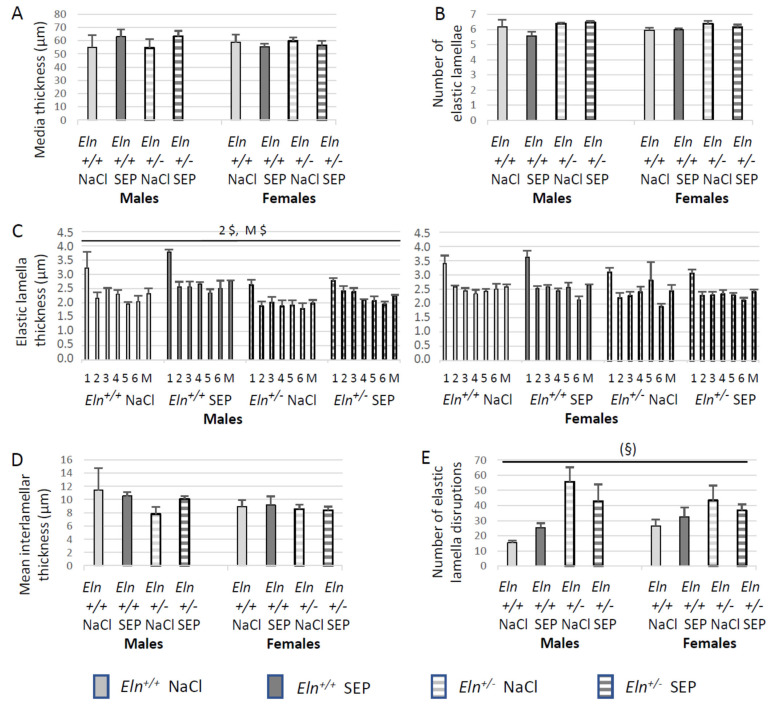
Analysis of the morphology and structure of the orcein-stained elastic fibers/elastic lamellae present in the distal part of the aortic arch, depending on genotype, sex and SEP treatment. The measurements were performed three days after NaCl (control) or SEP intravenous injection in the caudal vein of male and female mice of *Eln^+/+^* or *Eln^+/-^* genotypes. (**A**) Media thickness. (**B**) Number of elastic lamellae. (**C**) Mean thickness of the elastic lamellae (M) and thickness of each elastic lamellae, numbered from the luminal side of the media to the external side of the media, lamella #1 being the internal elastic lamina (see Figure 5 for illustration). (**D**) Mean of interlamellar thickness. (**E**) Number of elastic lamella disruptions. ^$^ significant interaction between sex and treatment (three-way ANOVA, *p* ≤ 0.05), showing that SEP treatment induced an increase in elastic lamella #2 thickness (2 $) or mean elastic lamella thickness (M $) in males, not females, independently of genotype (post hoc Fisher’s least significant difference (LSD) tests, *p* ≤ 0.05). ^(§)^ strong trend toward an interaction between genotype and SEP treatment, independently of sex (three-way ANOVA, *p* < 0.08), suggesting a lower number of elastic lamella disruptions in *Eln^+/-^* mice after SEP treatment. Control animals were injected with the same volume of 0.9% NaCl (NaCl). Values are mean ± SEM. n = 3–6 in each group.

Representative images of orcein-stained elastic fibers of aortae are presented in Figure 5. Histological analysis indicated that SEP treatment induced no significant difference between groups regarding the aortic media thickness, number of elastic lamellae and interlamellar thickness (Figure 6A,B,D). However, the aorta wall of SEP-treated mice presented an increase in mean lamella and lamella #2 thicknesses in male animals, not females, independently of the genotype (Figure 6C). SEP treatment also induced a strong trend (*p* < 0.08) toward a reduction in the higher rate of lamella disruptions naturally present in *Eln^+/-^* animals, independently of sex (Figure 6E). 

**Figure 7:**
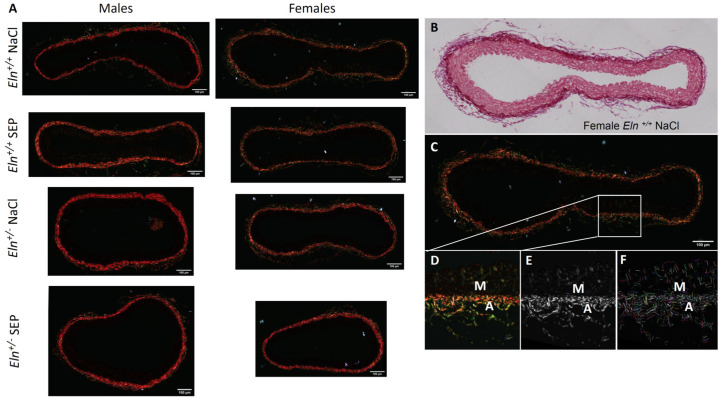
Images of picrosirius red-stained collagen fibers in cross-sections of the distal part of the aortic arch of *Eln^+/+^* or *Eln^+/-^* male or female mice injected with SEP or 0.9% NaCl (control), illuminated with polarized light (**A**). Representative example of an aorta from an *Eln^+/+^* female mouse injected with 0.9% NaCl (**B**–**F**), under illumination with non-polarized light (**B**), compared to polarized light (**C**–**F**). A magnified image of the selected area of the aorta wall shown in (**C**) is presented in (**D**). The images from the whole aorta wall were then CT-FIRE-processed in order to obtain the individual fiber properties and quantity of the collagen fibers in all the studied groups (fiber width, length, straightness, angle and number of fibers in the whole aorta section). The second harmonic generation (SHG) image generated from (**D**) using CT-FIRE program is shown in (**E**). The extracted fibers obtained using the CT-FIRE program are highlighted in different colors overlaid on the original SHG image in F. M = Media, A = Adventitia. Scale bars = 100 µm.

**Figure 8:**
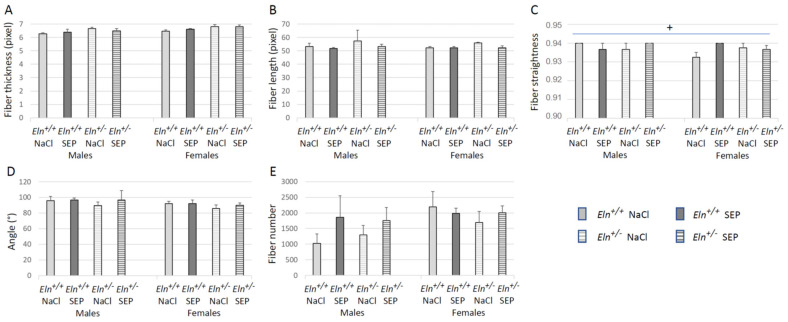
CT-FIRE analysis of picrosirius red-stained cross-sections of the distal part of the aortic arch (entire aorta wall section) illuminated with polarized light. The measurements were performed 3 days after NaCl (control) or SEP intravenous injection in the caudal vein of male and female mice of *Eln^+/+^* or *Eln^+/-^* genotypes. Individual properties and quantity of collagen fibers were compared between the studied groups: (**A**) fiber thickness, (**B**) length, (**C**) straightness, (**D**) angle and (**E**) number of fibers per aorta. ^+^ significant interaction between sex, genotype and SEP treatment (three-way ANOVA, *p* ≤ 0.05), showing that the elevation of collagen fiber straightness in untreated *Eln^+/+^* males, compared to *Eln^+/+^* female mice, was abolished by the SEP-induced increase in collagen fiber straightness in female *Eln^+/+^* mice (post hoc Fisher’s least significant difference (LSD) tests, *p* ≤ 0.05). Control animals were injected with the same volume of 0.9% NaCl (NaCl). Values are mean ± SEM. n = 3–6 in each group.

Morphometric and quantitative analyses (curvelet transform-fiber extraction, CT-FIRE) of picrosirius red-stained collagen fibers of aortae were then performed. Representative images are presented in Figure 7. No effect of SEP treatment could be observed on the collagen fiber thickness, angle and collagen fiber number per aorta section in the entire wall and media cross-sections (Figure 8A,D,E and Supplementary Materials Figure S2A,D,E) and length in the entire aorta wall only (Figure 8B). However, SEP treatment induced a collagen fiber length decrease in the aortic media of female *Eln^+/-^* and an increase in female *Eln^+/+^* mice, while no change could be detected in male animals (Supplementary Materials Figure S2B). The elevation of collagen fiber straightness found in the entire wall of untreated *Eln^+/+^* males, compared to *Eln^+/+^* female mice, was abolished by SEP treatment due to the SEP-induced increase in collagen fiber straightness in female *Eln^+/+^* mice, while no impact of SEP could be detected in *Eln^+/-^* animals (Figure 8C). No effect of SEP treatment on collagen fiber straightness could be observed when the analysis was restricted to the media (Supplementary Materials Figure S2C).

### 2.6. Aortic Tissue mRNA Levels

**Figure 9:**
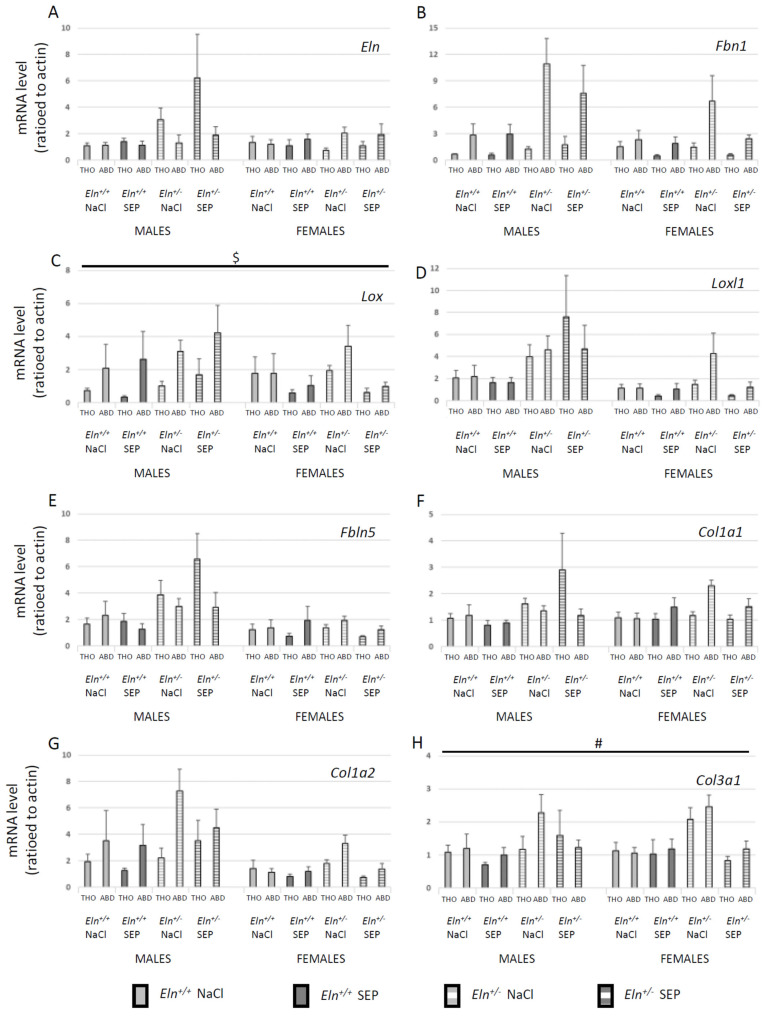
Effect of SEP treatment on the levels of mRNAs transcribed from the genes of the major components of aortic elastic and collagen fibers. The measurements were performed by RT-qPCR in thoracic (THO) and abdominal (ABD) aorta segments 3 days after SEP or NaCl (control) intravenous injection in the caudal vein of male and female mice of *Eln^+/+^* or *Eln^+/-^* genotype. (**A**) tropoelastin (*Eln*), (**B**) fibrillin-1 (*Fbn1*), (**C**) lysyl oxidase (*Lox*), (**D**) lysyl oxidase-like-1 (*Loxl1*), (**E**) fibulin-5 (*Fbln5*), (**F**) type I collagen alpha-1 (*Col1a1*), (**G**) type I collagen alpha-2 (*Col1a2*), (**H**) type III collagen alpha-1 (*Col3a1*). ^$^ significant interaction between sex and SEP treatment, independently of genotype and vessel segment (four-way ANOVA, *p* ≤ 0.05), indicating that SEP significantly decreased mRNA levels in female, not male, mice (post hoc Fisher’s least significant difference (LSD) tests, *p* ≤ 0.05). ^#^ general significant difference between untreated and SEP-treated mice, independently of sex, genotype and vessel segment (four-way ANOVA, *p* ≤ 0.05). Control animals were injected with the same volume of 0.9% NaCl (NaCl). Values are mean ± SEM. n = 5 in each group.

In order to have a wider view of the SEP impact on gene expressions in large elastic arteries and to save animals, the gene transcripts were measured in descending thoracic and abdominal aorta segments by RT-qPCR. First, the effects of SEP treatment were not significantly different between the thoracic and abdominal aorta segments (four-way ANOVA, 0.29 < *p* < 0.85, depending on the gene) (Figure 9). While SEP treatment had no impact on the *Eln*, *Fbn1*, *Loxl1*, *Fbln5*, *Col1a1* and *Col1a2* mRNA levels (Figure 9A,B,D–G), SEP significantly decreased the *Lox* mRNA level in female, not male, mice, independently of the genotype and vessel segment (Figure 9C), and it generally decreased the level of *Col3a1* mRNA (Figure 9H).

### 2.7. Aortic Tissue Protein Levels

**Figure 10:**
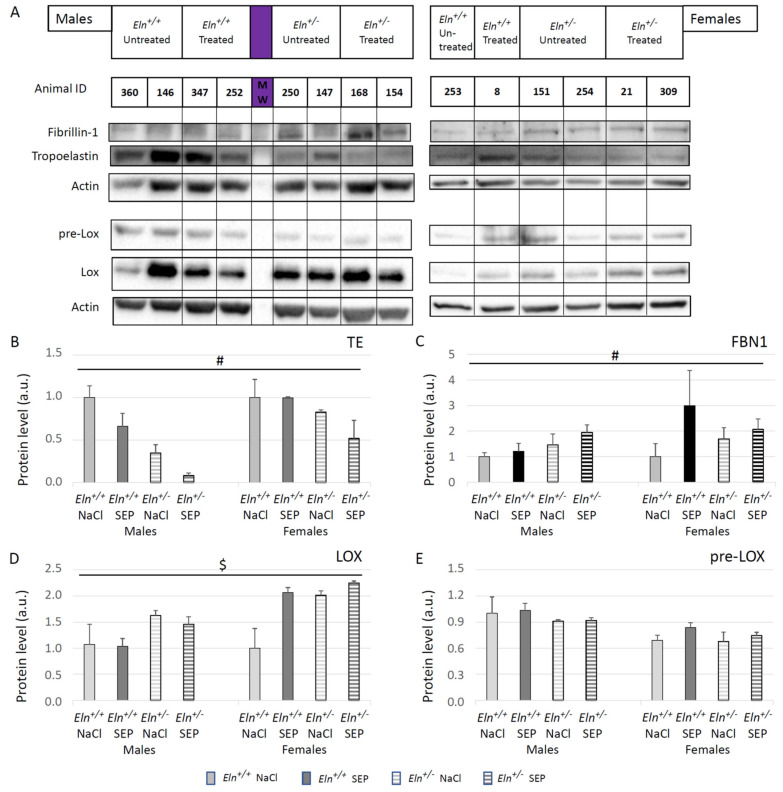
Western blotting of descending thoracic aorta extracts from untreated or SEP-treated mice. (**A**) Representative Western blot bands for the proteins of interest in each group. Semi-quantitative analysis of the protein levels was performed by band densitometry for: (**B**) tropoelastin (TE), (**C**) fibrillin-1 (FBN1), (**D**) lysyl oxidase (LOX, 30kDa), (**E**) immature lysyl oxidase (pre-LOX, 50kDa). ^#^ general significant difference between untreated and SEP-treated mice (three-way ANOVA, *p* ≤ 0.05). ^$^ significant interaction between sex and treatment, independently of genotype (three-way ANOVA, *p* ≤ 0.05), indicating that SEP elevated the protein level in females, not males (post hoc Fisher’s least significant difference (LSD) tests, *p* ≤ 0.05). Control animals were injected with the same volume of 0.9% NaCl (NaCl). Values are mean ± SEM. n = 5 in each group.

Western blotting of descending thoracic aorta extracts was performed for four major proteins involved in the elastic fiber structure or synthesis: TE, FBN1, LOX and immature LOX (pre-LOX). Representative images are presented in Figure 10A. SEP treatment generally decreased TE and elevated FBN1 levels (Figure 10B,C) compared to corresponding NaCl-treated controls, independently of the sex and genotype. Treatment with SEP also elevated LOX levels in females, not males, independently of the genotype, and it had no effect on pre-LOX levels (Figure 10D,E).

### 2.8. VSMC Proliferation and Tropoelastin Production

**Figure 11:**
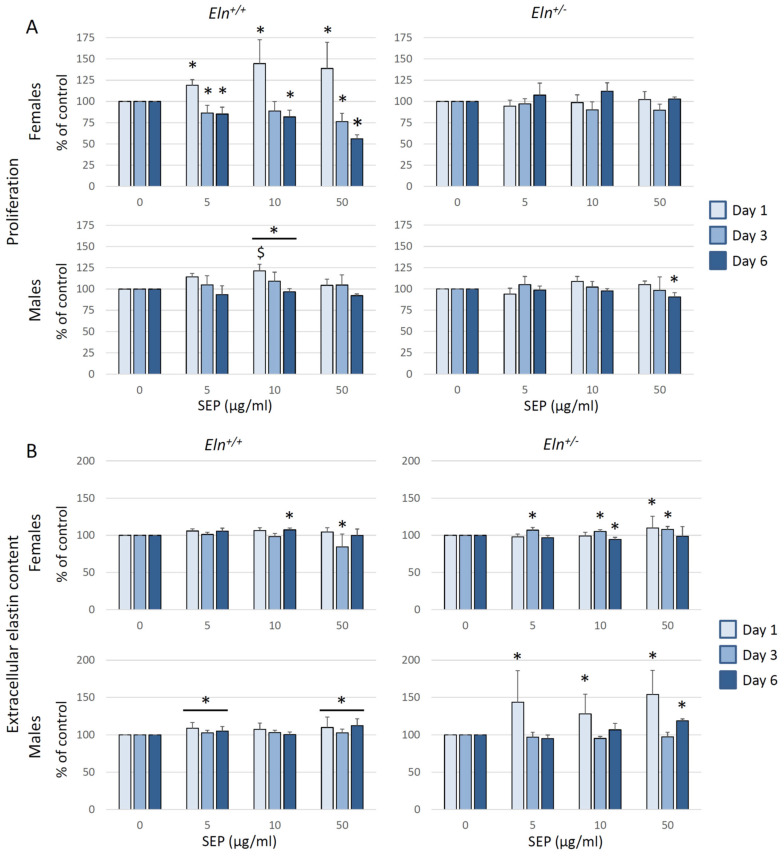
Proliferation and extracellular elastin content in primary cultures of aortic VSMCs from male or female *Eln^+/+^* or *Eln^+/-^* mice. VSMCs were treated with SEP (0, 5, 10 or 50 µg/mL) for 1, 3 or 6 days. (**A**) Impact of SEP treatment on VSMC proliferation, evaluated by using the MTT assay. The results are expressed as percent of the control value (0 µg/mL SEP) of the same day. (**B**) Impact of SEP treatment on extracellular elastin content, evaluated by using an ELISA assay. The results are expressed as percent of the control value (0 mg/mL SEP) of the same day. * significant difference with same day control (three-way ANOVA, followed by LSD test for paired comparisons, *p* ≤ 0.05). ^$^ significant difference at day 1 with same day control (two-way ANOVA, followed by LSD test for paired comparisons, *p* ≤ 0.05). Values are mean ± SEM. n = 3–5 primary cultures from separate animals in each group.

In order to evaluate the impact of SEP on the VSMC functions related to arterial mechanics and reactivity, we measured the cell proliferation and extracellular elastin level in primary cultures of aortic VSMCs of mice from each studied group. In *Eln^+/+^* VSMCs, SEP induced a clear 21% (males)–44% (females) increase in proliferation after one day of treatment, depending on the concentration, while SEP decreased (females) or had no impact on (males) VSMCs proliferation during the following days. This biphasic effect was weak (males) or absent (females) in *Eln^+/-^* VSMCs (Figure 11A).

After verification that the antibodies directed against elastin do not recognize SEP (Supplementary Figure S4), SEP was also found to induce an increase in extracellular elastin quantity in VSMC cultures from all four animal groups, although with marked variations depending on the genotype, number of days of treatment or SEP concentration. To be noted is the fact that, for a given mouse sex and genotype group, the time and SEP concentrations for which elastin overproduction was observed were those for which SEP induced no significant elevation of proliferation or a decreased proliferation of VSMCs. In females of both genotypes and *Eln^+/+^* males, the increase in extracellular elastin peaked in the range of +10%. In *Eln^+/-^* males, the elevation of extracellular elastin content was much higher and reached levels in the range of + 50% after 1 day of treatment. Only two exceptions were found in females (*Eln^+/+^*: day 3–50 µg/mL; *Eln^+/-^*: day 6–10 µg/mL), where the extracellular matrix content following SEP treatment was decreased by a few percentage points (Figure 11B).

## 3. Discussion

### 3.1. Differences between Eln^+/+^ and Eln^+/-^ Mice

In the present study, we first confirmed the already demonstrated morphologically, structurally and mechanically atypical characteristics of the heart and ascending aorta of 6-month-old *Eln^+/-^* mice compared to their wild-type counterparts [12,18,19,25]: cardiac hypertrophy, hypertension, narrower (and thinner walled for males), longer and generally stiffened ascending aortae, although more distensible at low pressure and less distensible at high pressure, as well as more numerous, thinner and more degraded elastic lamellae. These features of *Eln^+/-^* mice, previously demonstrated to be present in male animals, were also shown here to be present in females. In addition, an endothelium dysfunction—i.e., decreased relaxation in response to Ach—was associated with the *Eln^+/-^* genotype in females, not males, in accordance with the previously observed similar response to Ach in *Eln^+/+^* and *Eln^+/-^* 6-month-old male mice [12].

### 3.2. Impact of SEP Treatment on Blood Pressure and Heart

In this context, SEP treatment induced several modifications of the heart or aorta characteristics, with differential effects depending on the genotype or sex of the animals. 

In vivo, three days after the single intravenous injection of SEP, no change in blood pressure could be detected. However, SEP increased the inner and outer diameters of the ascending aorta in *Eln^+/+^* mice only and mildly elevated the total heart and left ventricle weights of males, not females, compared to controls and/or females, independently of the genotype. The sex-dependent effect of SEP on heart weight is consistent with previous results, showing that (i) cardiac hypertrophy is favored by the male hormone testosterone and inhibited by the female hormone estrogens [39] and that (ii) the injected elastin peptide binding to the ERC improves the heart rate–blood pressure product (= heart work) in male rats, in a NO-dependent manner (proven post-ischemia) [40], which correlates with the left ventricle mass [41]. The moderate elevation of the heart weight observed after SEP treatment resembles the cardiac hypertrophy induced by the elastin production stimulator minoxidil in male rats and mice [26,27,28].

### 3.3. Impact of SEP Treatment on Aortic Structure and Mechanics

First, in vitro, SEP increased the proliferation of aortic VSMCs from *Eln^+/+^*, not *Eln^+/-^*, mice at day 1 following treatment, while it limited cell proliferation in the following days, the latter also being true in male *Eln^+/-^* mice.

SEP also stimulated the production of elastin by cultured aortic VSMCs from male and female *Eln^+/+^* and *Eln^+/-^* mice, although with a substantially higher amplitude in male *Eln^+/-^* animals. Consistently, in vivo, SEP treatment increased the elastic lamella thickness in aortae from male, not female, animals of both genotypes. Taken together, these results suggest that the SEP-induced elastic lamella thickening could be due to elastic fiber neosynthesis, possibly accompanied by SEP integration in elastic lamellae. Both hypotheses are consistent, at least in males, with the results related to the mechanics of the ascending aorta, which showed that the naturally increased stiffness of *Eln^+/-^* aortae is reduced by SEP and returns closer to the stiffness of aortae from *Eln^+/+^* animals. This is similar to the effect induced by minoxidil, shown in vivo to induce elastic fiber neosynthesis and aortic elasticity improvement together with cardiac hypertrophy in male rats and mice [25,26,27,28]. In addition, while the elastic lamellae from untreated *Eln^+/-^* animals presented substantially more disruptions than those from *Eln^+/+^* mice, a strong trend toward the abolition of this difference was observed following SEP treatment. Hence, this also suggests the potential of SEP for repairing the pre-existing elastic fibers, which matches the already shown protective/restorative effect on elastic fibers of minoxidil and dill extract, two stimulators of elastic fiber synthesis [25,27,28,32].

Of particular interest is the fact that SEP treatment also modified the conformation of aortic collagen fibers in female, not male, mice. In female *Eln^+/+^* animals, SEP injection increased the collagen fiber length (in the media) and straightness (in the entire wall), reaching the higher straightness value found in males. Therefore, this could not explain the SEP-induced increases in outer and inner diameters observed in *Eln^+/+^* mice of both sexes. In the aorta media of female *Eln^+/-^* mice, SEP treatment decreased the collagen fiber length, which has to be put in perspective with the impact of SEP treatment on the aortic mechanical and morphological properties observed in *Eln^+/-^* animals. In *Eln^+/-^* mice, the SEP-induced decrease in aortic stiffness was lower in females than males, which could partly be due to the SEP-induced decrease in collagen fiber lengths detected in *Eln^+/-^* females, not males.

### 3.4. Mechanisms of Action of SEP on Aortic Structure and Mechanics: Facts and Hypotheses

In order to better understand the mechanisms allowing SEP to improve aortic elasticity, we studied the tissular production of a series of gene transcripts related to collagen or elastic fibers and measured the level of the major proteins involved in elastic fiber synthesis, i.e., tropoelastin, fibrillin-1 and lysyl oxidase. In aortic segments, SEP treatment significantly decreased the *Lox* mRNA expression level in female, not male, mice of both genotypes. This effect, observed at day 3 post-SEP injection, is consistent with the absence of elastic lamella thickening in these animals, although it is concomitant with the increased LOX protein content at the same time. The latter possibly results from an earlier peak of protein production and could lead to a later elastic fiber synthesis. Further supporting the positive impact of SEP on elastic fiber neosynthesis in the mouse aorta, fibrillin-1 production was found generally elevated by SEP treatment, while, surprisingly, tropoelastin was decreased, independently of the sex or genotype. This could indicate that, at the particular timepoint chosen in our study (3 days after SEP injection), the tissular tropoelastin stocks have been used for integration in the newly synthesized elastic fibers, with no stimulation of new tropoelastin production (as suggested by the qPCR experiments) and a simultaneous overproduction of fibrillin-1. In parallel, SEP treatment induced no change in the *Col1a1* and *Col1a2* mRNA levels, as well as a general reduction in the *Col3a1* mRNA expression level, independently of the sex and genotype. The SEP-induced modifications of collagen type distribution and conformation could also account for the structural and mechanical changes observed in the aorta. This is supported by previous results, i.e., (i) changes in type I/type III collagen ratio and collagen fiber lengths correlate with a switch from normal to disease state in tissues, as shown in the heart [42], and (ii) type I collagen synthesis/degradation ratio negatively correlates with arterial stiffness [43], suggesting that a SEP-induced increase in type I/type III collagen ratio could contribute to the observed decreased aortic stiffness, in particular in *Eln^+/-^* animals.

The specificities of the aortic VSMCs from Eln^+/-^ mice could also be involved in the modulation of arterial or cell functions by SEP, since SEP induced differential effects in *Eln^+/+^* and *Eln^+/-^* mice regarding VSMC reactivity, proliferative and synthetic functions. It was already demonstrated that VSMCs from *Eln^-/-^* and *Eln^+/-^* mice produce no (*Eln*^-/-^) or less (*Eln^+/-^*) elastin [12,18,19,44] and spontaneously hyperproliferate compared to VSMCs from *Eln^+/+^* animals. A similar coincidence between low elastin production and hyperproliferation was also observed in cultured VSMCs from SVAS or WBS patients [45]. *Eln^-/-^* VSMCs return to a normal growth rate after the addition of tropoelastin, and *Eln^+/+^*, *Eln^+/-^* and *Eln^-/-^* VSMC proliferation is strongly reduced by rapamycin, an antiproliferative mTOR inhibitor [46,47]. Here, we showed that SEP is able to stimulate the proliferation of cultured VSMCs in *Eln^+/+^* mice early after treatment (day 1) and then to reduce it (days 3–6) in *Eln^+/+^* mice of both sexes and male *Eln^+/-^* animals. SEP also stimulated elastin synthesis by VSMCs at various time points and SEP concentrations, depending on the animal group, although especially in *Eln^+/-^* male mice, which is the group in which SEP significantly decreases arterial stiffness. The long-term (days 3–6) inhibition of VSMC proliferation by SEP resembles the previously demonstrated growth inhibition by tropoelastin of VSMCs from elastin-deficient mice 3 days after treatment [46]. Additionally, of importance is the fact that, for a given mouse sex and genotype group, the times and SEP concentrations for which elastin overproduction by VSMCs was observed were those for which SEP induced no significant elevation of proliferation or a decreased proliferation of these cells. This is consistent with previous observations showing an inverse effect of elastin peptides on chick VSMC proliferation (increase) and elastin production (decrease), probably due to the regulation of elastin production by/during cell growth [48]. These authors suggested that this effect could be mediated by the binding of elastin peptides to the elastin receptor (now known as the elastin receptor complex, ERC). The results reported here could be related to the same mechanism, since SEP includes binding sequences to the ERC, as shown by the inhibition of fibroblast adhesion onto SEP-coated surface by ERC blockade (patent #WO2017194761) [35,37,49,50]. Actually, SEP was designed to contain the same binding sites as tropoelastin regarding ERC and integrin αvβ3, the binding of tropoelastin to integrin αvβ3 inducing cell adhesion and spreading [34]. This makes it possible/likely that one or both of these receptors is/are involved in the mechanism of action of SEP.

### 3.5. Impact of SEP Treatment on Aortic Reactivity

In addition to its differential impact, depending on the genotype, on VSMC proliferation and elastin production, SEP also acted in a genotype- and sex-dependent manner on arterial reactivity. SEP treatment increased the contractile response of the ascending aorta to acute phenylephrine (Phe, ≥10^−7^ M) in female animals of both genotypes and male *Eln^+/+^* mice only. Additionally, SEP treatment restored to normal the altered vasodilatory response of female *Eln^+/-^* aortae to acute acetylcholine (Ach, ≥ 10^−8^ M), characterizing an endothelial dysfunction in these animals, and also improved the vasodilatory response of male *Eln^+/-^* aortae to 10^−7^ M acetylcholine only. These effects indicate a rather general SEP-induced improvement of the aortic motricity. However, the absence of enhancement by SEP of the Phe-induced vasoconstriction in male *Eln^+/-^* animals is puzzling and might be related to a particular regulation of the α-adrenoceptor pathway by both sex/sex hormones and genotype, as previously suggested by the absence of age-dependent decline in Phe-induced vasoconstriction in male *Eln^+/-^* mice [12]. In the other three groups, the augmented Phe-induced aortic constriction following SEP treatment could be related to the SEP amino-acid sequences derived from tropoelastin, which are the binding sites for the ERC present at the membrane of VSMCs [35]. First, it was shown that tropoelastin application to cultured *Eln^-/-^* VSMCs promotes the contractile phenotype of these cells by inducing the formation of organized actin stress fibers through the mediation of the tropoelastin sequence VGVAPG, known to bind to the ERC [46,51]. This phenomenon could additionally be enhanced in the aorta of *Eln^+/-^* animals—here, in females, which undergo a substantially lower circumferential stress and higher stretch (or strain), since VSMCs are known to be integrin-mediated mechanical sensors in the arterial wall and regulate the actin network organization, i.e., contractile capability, as a function of wall stretch and stress [38]. The altered vasodilatory response to Ach in female *Eln^+/-^* mice only, possibly related to an interaction between the genotype and female hormones, is consistent with previous results showing no differential vasodilatory response to the NO-dependent vasodilator Ach between the ascending aortae from male *Eln^+/+^* or *Eln^+/-^* mice and a hyperconstricted state of renal arteries in female *Eln^+/-^* mice only [12,19,52]. Since a similar vasodilatory response to the NO donor SNP, acting directly on VSMC relaxation, was observed in the four animal groups, it could be concluded that an endothelial dysfunction explains the lower response of the ascending aorta of female *Eln^+/-^* mice to Ach. The restoration of a normal vasodilatory response of these animals to Ach after SEP treatment could be explained by the already demonstrated endothelium- and NO-dependent vasodilatory action of elastin sequences binding to the ERC [53,54,55,56], since some of these sequences, including VGVAPG, are present in SEP.

### 3.6. Sex-Related Differences in the Response to SEP Treatment

Of particular interest are the different responses of male and female animals to the treatment with SEP. First, after inducing an initial over-proliferation of VSMCs from *Eln^+/+^* mice of both sexes, SEP treatment induced a delayed reduction in VSMC proliferation in females only, while virtually no sex-related difference could be detected in *Eln^+/-^* animals. Additionally, compared to the effects observed in female animals, SEP elevated substantially more the elastin production by VSMCs from *Eln^+/-^* males and induced a thickening of the elastic lamellae (#2 and mean lamella thickness) in males, independently of the genotype. This is of particular importance, since the SEP-induced decrease in aortic stiffness was of greater amplitude in male mice compared to females. This biomechanical difference is probably also related to the other SEP-induced structural changes observed in females, not males, at day 3 post-treatment, including decreased *Lox* mRNA and increased LOX protein levels (independently of the genotype), as well as increased collagen fiber straightness and length in *Eln^+/+^* mice and decreased collagen fiber length in *Eln^+/-^* animals. Unexpectedly, in *Eln^+/-^* mice, SEP also enhanced the Phe-induced vasoconstriction and Ach-induced vasodilation in females only, with the only exception of improved vasodilation in male *Eln^+/-^* animals at 10^−7^ M Ach.

Such sex-related differences in response to SEP treatment are not surprising since, as indicated above, sex hormones have been shown to interfere with several pathophysiological mechanisms, such as cardiac hypertrophy, which is favored by the male hormone testosterone and inhibited by the female hormone estrogens [39]. A differential structural and functional impact of pharmacological treatment with minoxidil (an elastin production inducer) on arteries has also been demonstrated as a function of sex. Minoxidil was shown, in the abdominal aorta, to increase the elastin and collagen contents, decrease stiffness in females, not males, and elevate the inner diameter in males, not females [28]. Therefore, future trials should expect differential effects of SEP in males and females, which may be of importance to anticipate the precise adaptation of possible future treatments to the animal or patient sex.

### 3.7. Questions about the Presence of SEP in the Aorta Wall and Conclusions

We also tried to identify the presence of SEP in the aorta wall or, more precisely, in the elastic lamellae, in order to verify the possibility of improving arterial mechanics by direct integration of SEP in the wall or elastic lamellae. Unfortunately, the commercial antibodies to the SEP flag tag were found inoperative in immunofluorescence histology or Western blotting of mouse aortic tissues, since they revealed a supposed “SEP flag” signal in both SEP-treated and untreated animals. The design of a new strategy for SEP identification in the aortic wall will therefore be necessary, possibly by generating and injecting biotin-coupled SEP in mice. Without excluding the possibility that direct SEP integration in the aortic wall and elastic fibers could occur and participate in the observed effects, we therefore favor the second hypothesis of cellular SEP signaling to explain the SEP-induced changes in the aortae of the studied animals.

Specifically, regarding the effects of SEP in the aorta of *Eln^+/-^* mice, our results show a generally improved/decreased stiffness, although of higher amplitude in males, probably due to two kinds of mechanisms. First, mechanisms common for both sexes could be involved, i.e., decreased elastic lamella disruption and possibly increased type I/type III collagen ratio. Second, more sex-specific mechanisms could also contribute, such as elastin production and elastic lamella thickening, mainly present in males, or a decrease in collagen fiber length, mainly present in females. The abolition by SEP of the altered Ach-induced vasodilation specifically present in female mice is also likely to be beneficial for the altered hemodynamics. These elements are of importance in view of the possible future treatments of SVAS/WBS patients (and possibly cutis laxa patients), a high proportion of whom present cardiovascular abnormalities similar to those observed in *Eln^+/-^* mice, such as hypertension, altered arterial elastic lamellae/fibers, cardiac hypertrophy or increased arterial stiffness. Treatments have to be as individualized as possible, and here, they could rely at least on sex-specific targeting of molecular effects or organ function improvements. The potential SEP treatment of patients could then be expected to be efficient regarding the improvement of the arterial elastic lamella/fiber alterations, especially in men, and elevated stiffness in both sexes, with no beneficial impact on the elevated blood pressure and a need for vigilance in male patients, since SEP treatment induced a mild cardiac hypertrophy in male animals, similar to that produced by minoxidil [27,28]. Since our in vivo results were obtained after a single injection of SEP only, future studies should verify whether a higher dose and/or multiple injections of SEP could induce an even better amelioration of the arterial function. However, our results already suggest that SEP should be considered as a tool with an interesting potential in future trials aiming at the improvement of the arterial function in human conditions related to genetic elastin deficiency.

## 4. Materials and Methods

### 4.1. Animals

Two hundred and twenty-one 6-month-old male and female mice (animal age between 5 and 7 months old), wild-type (*Eln^+/+^*) or bearing a heterozygous deletion of exon 1 in the elastin gene (*Eln^+/−^*, which leads to one non-functional *Eln* allele), were backcrossed for more than five generations into the C57B1/6J background and used in these experiments [18,44]. The animals were distributed into 8 groups: male or female, *Eln^+/+^* or *Eln^+/-^*, treated with SEP or untreated (control, injected with NaCl). A total of 80 animals were used to perform aortic mechanics studies, body weight/heart weight/hematocrit measurements, tissular mRNA and protein level evaluation (10 for each of the 8 groups); 80 more were used for vasoreactivity experimentations and histology (10 for each of the 8 groups); then, 43 more animals were used for blood pressure tests (5–6 for each of the 8 groups). 

In addition, eighteen mice were distributed into 4 groups (male *Eln^+/+^*, female *Eln^+/+^*, male *Eln^+/-^*, female *Eln^+/-^*) and used for primary cultures of aortic VSMCs.

All animals were randomly housed in controlled conditions (temperature 21 °C ± 1 °C, humidity 60% ± 10%, lighting from 8:00 a.m. to 8:00 p.m.) and received a standard chow and tap water ad libitum.

All housing and experimental procedures were in accordance with institutional guidelines. The animal study protocols were approved by (1) the local Ethics Committee of the Université Grenoble Alpes (blood pressure measurements and all organ collections, except for tension arteriography and histology), (2) the local Ethics Committee of the Federative Structure of Research SFR BioSciences Lyon CNRS UMS3444/US8 (CECCAPP, C2EA15) (for organ collections for tension arteriography and histology) and by (3) the French ministry of research under the APAFiS numbers #20023-2019032809004121 v2 (4 July 2019) and #25475-2020050405428113_v2 (27 May 2020), respectively.

### 4.2. SEP Primary Structure and Production

SEP is a long polypeptide comprising 593 amino acids. It is organized as alternating hydrophobic and AK-rich cross-linking domains that mimic the native tropoelastin primary structure, preceded by a FLAG tag in its N-terminal region, as follows:

NH2CDYKDDDDK [(VGVAPGVGVLPG)6AAAKAAAKAAK]6 (VGVAPGVGVLPG)6-GGACLGKACGRKRK-COOH. The corresponding DNA coding sequence synthesis was outsourced to Genescript (Rijswijk, The Netherlands). The sequence was cloned in the prokaryotic expression vector pET-30a between NdeI and SalI restriction sites. The *E.coli* BL21 (DE3) strain was transformed with the pET-30a-SEP construct under kanamycin selection. Bacteria were precultured in Luria-Bertani broth supplemented with 50 µg/mL kanamycin (Sigma-Aldrich, Saint-Quentin-Fallavier, France), and the production was conducted in 2 L of Luria-Bertani-based auto-induction medium (Formedium, Hunstanton, UK) supplemented with 50 µg/mL kanamycin for 8 h/37 °C/150 rpm. Bacteria were centrifuged 4000× *g*/ 4 °C/ 20 min, and the pellet was frozen overnight at −80 °C. The pellet was thawed and resuspended in distilled water prior to mechanical cell disruption (Cell disruptor TS2, Constant Systems, Daventry, UK). The lysate was centrifuged at 10,000× *g*/4 °C/10 min, and the supernatant was further processed for SEP purification by the inverse transition cycling (ITC) method, as described by Meyer and Chilkoti [57]. Briefly, lysate was buffered with 50 mM Tris-HCl pH 8.8, and 0.2% polyethylenimine (Sigma-Aldrich) was added under agitation for 30 min/4 °C. The precipitate was discarded by centrifugation 15,000× *g*/4 °C/10 min. The supernatant was warmed to 40 °C for 10 min with 1 M NaCl prior centrifugation at 15,000× *g*/40 °C/5 min. The supernatant was discarded, and the pellet containing SEP was resuspended in cold buffer on ice for 10 min to end the first ITC. Three rounds of ITC were applied. The final pellet was resuspended in 0.9% NaCl. Quality controls were conducted by silver staining of sodium dodecyl sulfate-polyacrylamide gel electrophoresis (SDS-PAGE), bicinchoninic acid protein assay (ThermoFisher Scientific, Les Ulis, France), and the presence of endotoxin was quantified by limulus amebocyte lysate testing (Endosafe, Charles River, Ecully, France). The purified SEP concentration was adjusted to 10 mg/mL in 0.9% NaCl and kept frozen at −20 °C before use.

### 4.3. SEP Administration

After animal weighing, a single injection of 100 µL 0.9% NaCl (control) or SEP solutions was performed in the caudal vein of each animal. The injected SEP quantity was 12 μg/g. Three days after NaCl or SEP injections, all the mice were anesthetized by intraperitoneal injection of a ketamine (100 mg/kg)/xylazine (10 mg/kg) solution and heparinized (150 UI) by injection in the saphenous vein before organ collection and study by using the following methods. Another procedure, described below, was followed only in mice in which blood pressure was measured three days after saline or SEP injection.

### 4.4. Body Weight 

All the animals were weighed by using an adapted scale (precision: 0.1 g) three days after saline or SEP injection. 

### 4.5. Surgical and Post-Surgical Procedures

In order to optimize the use of the animals, we used different aorta segments depending on the technique: ascending aorta (mechanics and reactivity), distal part of the aortic arch between the left subclavian artery and the beginning of the descending thoracic aorta (histology), descending thoracic aorta (Western blots), descending and abdominal aorta (RT-qPCR) and entire aorta (cell cultures).

#### 4.5.1. Blood Pressure

In total, 43 animals, distributed into the 8 groups described above (5 per group), were studied for arterial blood pressure. Mice were anesthetized by isoflurane inhalation and had a heart rate of approximately 400–450 beats per minute (bpm). Isoflurane was administered with a vaporizer (MSSVAP02, Medical Supplies and Services International limited, Keighley, UK). After induction with 2.5–3.5% isoflurane in an isolated chamber, mice were placed on a heating table to maintain body temperature at 37 °C. Anesthesia was maintained with 1–1.5% isoflurane (with mix air/O_2_ flow 0.5/0.5 L/min) delivered through a small nose cone. A polyethylene catheter filled with a heparinized saline solution connected to a pressure probe and an acquisition system Powerlab 26T (AD instruments, Dunedin, New Zealand) and acquisition software Labchart (AD instruments) was inserted into the left carotid artery. Systolic, diastolic and mean arterial blood pressures, as well as heart rate, were measured for a period of time (2–5 min) during which the signal was stable.

#### 4.5.2. Heart Weight and Hematocrit

Under anesthesia, the hearts were then collected, washed and weighed (wet weight). The left ventricle and septum were then dissected, washed and separately weighed (wet weight). Total heart weight to body weight (HW/BW) and left ventricle + septum weight to body weight (LV + S/BW) ratios were calculated as percentages. During this procedure, some blood from each animal was collected in hematocrit tubes, and the hematocrit was read after tube centrifugation.

#### 4.5.3. Cannulated Ascending Aorta Mechanics—Pressure Arteriography

The ascending aorta was excised and placed in a physiological buffer composed of 135 mM NaCl, 5 mM KCl, 1.6 mM CaCl_2_, 1.17 mM MgSO_4_, 0.44 mM KH_2_PO_4_, 2.6 mM NaHCO_3_, 0.34 mM Na_2_HPO_4_, 5.5 mM D-glucose, 0.025 mM EDTA, 10 mM HEPES (pH 7.4). The vessels were then cannulated and mounted onto a pressure myograph placed under a video microscope, allowing for aorta bathing and filling with the physiological solution at 37 °C. A proprietary video analysis software system, WinDiam, coupled to the video microscope, was used to measure the inner (ID) and outer (OD) vessel diameters while changing the intraluminal pressure of the vessel, i.e., changing the pressure of the physiological buffer filling the vessel lumen from 0 to 175 mmHg [12,58]. Below 125 mmHg, where the ascending aorta wall is too thick to allow for accurate measurement of the transilluminated vessel ID, ID was calculated as described in Ref [58]. Distensibility, i.e., the relative change in luminal volume (percentage) per mmHg, was then calculated. Here, we used the distensibility per 25 mmHg increment (D25). The circumferential midwall strain (ε), circumferential wall stress (σ) and incremental elastic modulus (Einc) were calculated according to classical formulae [59]. ε is the relative increase in diameter at a given pressure compared to the diameter at no pressure. σ are the forces, which are circumferentially applied on each small portion of the vessel wall. Einc is indicative of wall stiffness. The detailed protocol is described in Appendix A.1.

#### 4.5.4. Ascending Aorta Length, Ring Reactivity and Mechanics—Tension Arteriography

Mice were anesthetized with ketamine (200 mg/kg, i.p.) and xylazine (20 mg/kg, i.p.). The ascending aorta (from the sinotubular junction of the aortic root to brachiocephalic artery) was removed rapidly and placed in a cooled physiological saline solution (PSS) bubbled with 95% O_2_ and 5% CO_2_. After cutting and measuring the length by using a digital microscope, the vessels were transferred into the chambers of a Multi Wire Myograph (Model 610M; Danish Myo Technology A/S, Aarhus, Denmark) filled with PSS at 37 °C (pH 7.4). The aorta ring was mounted onto two pins (200 μm diameter). The effective pressure was normalized by the stretching of the aorta, reaching a tension corresponding to the target pressure of 100 mmHg (IC100). Then, the aorta was set to a distension corresponding to 90 mmHg (90% of IC100). At this resting pressure, the aortic rings were pre-contracted with 60 and 120 mM KCl, successively [60]. After a 30 min equilibration period at resting pressure, the cumulative concentration–response curves of phenylephrine (Phe, 1 × 10^−9^ to 3 × 10^−6^ M), acetylcholine (Ach, 1 × 10^−8^ to 3 × 10^−5^ M) and sodium nitroprusside (SNP, a NO donor inducing endothelium-independent and VSMC-dependent vasodilation, 1 × 10^−9^ to 3 × 10^−6^ M) were consecutively studied, as previously described [60]. Vasoconstriction was expressed as the changes of tension (ΔmN/mm vessel length). Relaxation was evaluated in vessels pre-contracted with Phe inducing 40−60% of maximal vasoconstriction and was calculated as the percentage of reversal of the Phe-induced vascular tone. At the end of the vascular reactivity study and after a 15 min period of equilibration, the aortic rings were gradually stretched by 200 µm steps using a micrometric expender. In response to stretching, the internal circumference increased, leading to an elevation of transmural pressure, expressed in kPa.

#### 4.5.5. Histological Staining and Image Analysis

For histological analyses, the distal part of the aortic arch (between the left subclavian artery and the beginning of the descending thoracic aorta) was fixed with formalin and embedded in paraffin (FFPE), then cut into 5 µm thick sections. Staining was performed after dewaxing and rehydration up to water.

##### Elastic Fiber Staining—Orcein Acid (According to Shikata’s Method)

Orcein staining was carried out by immersing aortic sections for 30 min in a commercial solution of acid orcein (#010251, Diapath, Martinengo, Italy) [61,62]. The slides were then rinsed for 5 min in different solutions (70% EtOH, then distilled water, 100% EtOH for 5 min, EtOH/HCl (99.5/0.5, *v*/*v*) and then running tap water). Counterstaining was then performed for 5 min using Hematoxylin counterstain solution (#H-3401-500, Vector Laboratories, Newark, CA, USA), and the sections were then rinsed in running tap water. No counterstaining was performed in the sections used for elastic lamella number and disruptions in order to facilitate the quantifications. After dehydration, sections were mounted in an organic mounting medium (BioMount DPC, Biognost, Zagreb, Croatia) and scanned using the AXIO SCAN.Z1 slide scanner (Zeiss, Rueil Malmaison, France).

##### Collagen Fiber Staining—Picrosirius Red

Rehydrated tissue sections were stained for 1 h with Sirius Red F3B (Direct Red 80, #365548, Sigma-Aldrich, Saint Quentin-Fallavier, France) solution at 1 g/L in saturated aqueous solution of picric acid (1.3%). After 2 quick washes in 0.5% acidified water, the sections were dehydrated, mounted and scanned using the AXIO SCAN.Z1 slide scanner (Zeiss) under brightfield and linear polarized filters, as birefringence under polarized light is highly specific for collagen [63]. The birefringent images of the aorta sections, captured at 90°, were analyzed through the curvelet transform–fiber extraction (CT-FIRE) analysis for fiber length, thickness, density, straightness, angle and alignment between collagen fibers using the CT-FIRE V3.0 Beta software, as described previously [64,65,66,67].

#### 4.5.6. mRNA Level Analyses—RT-qPCR

##### DNA and RNA Extraction

For RNA extraction, abdominal and descending thoracic aortae were homogenized in TRIzol Reagent (#15596026, Invitrogen, Carlsbad, CA, USA), and RNA was extracted after precipitation with isopropanol and ethanol washes according to the manufacturer’s protocol. RNA samples were stored at −80 °C after flash-freezing in liquid nitrogen.

##### Reverse Transcription and Quantitative Real Time PCR Analysis

One microgram of RNA was reverse-transcribed to cDNA using the iScript complementary DNA (cDNA) synthesis kit (#1708840, Bio-Rad Laboratories, Hercules, CA, USA), according to the manufacturer’s protocol. cDNA or DNA were used for quantitative real-time PCR (RT-qPCR) using the SsoAdvanced SYBR Green Supermix kit (#1725270, Bio-Rad Laboratories), according to the manufacturer’s protocol. Gene expression was normalized to beta actin. The genes of interest were: *Eln* (tropoelastin), *Fbn1* (fibrillin-1), *Lox* (lysyl oxidase), *Loxl1* (lysyl oxidase-like-1), *Fbln5* (fibulin 5), *Col1a1* (type I collagen alpha-1), *Col1a2* (type I collagen alpha-2), *Col3a1* (type III collagen alpha-1). The primer sequences used for analyses are listed in Appendix A.2.

#### 4.5.7. Western Blots

A snap-frozen descending thoracic aorta was homogenized in 200 µL RIPA buffer (Tris HCl, pH 8 (50 mM) final, NaCl (150 mM) final, NP40 (1%) final, DOC (sodium deoxycholate) (0.5%) final, SDS (0.1%) final, Proteases inhibitor 1× final, PMSF (1 mM) final) and lysed using ceramic beads with a Precellys 24 (6000 rpm, 3 × 10 s) or 300 µL RIPA buffer in Polytron to extract the proteins. The lysate was centrifuged at 9500× *g* at 4 °C for 10 min, and the supernatant was centrifuged again at 9500× *g* at 4 °C for 10 min before the collection of the supernatant. The protein concentration was calculated by using a Bradford assay using bovine serum albumin (BSA) as a standard, according to the manufacturer’s protocol (Bradford reagent, #5000006, Bio-Rad, Marnes-la-Coquette, France). Amounts of 15−20 µg of proteins were separated by electrophoresis on 8% (fibrillin-1, tropoelastin) or 12% (lysyl oxidase) SDS polyacrylamide gels for 20 min at 80 V, then for 1 h 20 min–2 h at 120 V, and transferred to polyvinylidene difluoride membranes and nitrocellulose membranes. Next, the membranes were blocked for 1 h at room temperature with 5% nonfat milk (tropoelastin) or 5% BSA (fibrillin-1, lysyl oxidase) in TRIS-buffered saline (TBS) with Tween 20 (0.1%). Membranes were then incubated overnight at 4 °C with the following primary antibodies in TBS-Tween 20–1% nonfat milk or BSA: anti-tropoelastin (1/1000, PR385, Elastin Products Company, Owensville, MO, USA), anti-fibrillin-1 (1/1000, Ab53076, Abcam, Cambridge, UK), anti-lysyl oxidase (1/1000, D8F2K, Cell signaling, Danvers, Massachusetts). Membranes were then incubated for 1 h at room temperature with the appropriate horseradish peroxidase-conjugated anti-IgG (anti-rabbit 1/5000, #111-035-003, Jackson ImmunoResearch, West Grove, PA, USA) before and after three washes in TBS, Tween 0.1%. The proteins were visualized by enhanced chemiluminescence with the Western Blot ECL substrate (Clarity, Bio-Rad) on a ChemiDoc analyser (Bio-Rad). The relative amount of protein was quantified by densitometry (Image Lab, Bio-Rad) and expressed as a ratio of the actin content.

### 4.6. Proliferation and Elastin Production by Cultured VSMCs 

#### 4.6.1. Primary Cell Cultures

As previously described, VSMCs were isolated from the entire aorta of eighteen mice (*Eln^+/+^* and *Eln^+/-^*, males and females). For each cell culture, one aorta was enzymatically digested with collagenase type 2 (1 mg/mL) for 30 min at 37 °C, 5% CO_2_. The adventitia was then mechanically removed, and the remaining medial + intimal tube cut in 2 mm long pieces was digested with collagenase type 2 (1 mg/mL) and elastase (0.5 mg/mL) for 40 min at 37 °C, 5% CO_2_, before mechanical trituration by continuous flushing with a pipetter for 10 min. The suspension was centrifuged at 200× *g* for 10 min, and the cells were collected and seeded in a well from a 24-well plate in Dulbecco’s modified Eagle’s medium (DMEM), containing 20% bovine fetal serum (FBS), 1% (*v*/*v*) penicillin/streptomycin solution and 1% non-essential amino acids solution (NEAA), and maintained in 5% CO_2_ humidified air at 37 °C. At sub-confluency, cells were isolated by trypsinization, and the cell culture was amplified and used at the 6th or 7th passage. 

#### 4.6.2. VSMC Proliferation—MTT Assay

The MTT assay was used to measure cellular metabolic activity as an indicator of proliferation. This colorimetric assay is based on a reduction of yellow tetrazolium salt (3-(4,5-dimethylthiazol-2-yl)-2,5-diphenyltetrazolium bromide or MTT) to purple formazan crystals by the metabolically active (= living) cells only. The viable cells contain NAD (P)H-dependent oxidoreductase enzymes, which reduce the MTT to formazan. VSMCs were cultured in 96-well plates in fresh 20% FBS-DMEM supplemented with 0, 5, 10 or 50 µg/mL SEP. Because in cell cultures, SEP does not potentially undergo a loss of potency due to in vivo metabolization, the SEP concentrations used for cultured VSMC treatment were lower than those used for intravenous injection in mice. After 1, 3 or 6 days, the cells were incubated for 4 h at 37 °C, 5% CO_2_ with 15 µL Dye solution (cat. #G4101, Promega, Madison, WI, USA). Then, the insoluble formazan crystals were dissolved using 100 µL solubilization solution (cat. #G4102, Promega, Madison, WI, USA) for 1 h at room temperature; then, they were homogenized using a pipette, and the resulting colored solution was quantified by measuring the absorbance at 560 nm with a multi-well spectrophotometer (1681000, Bio-Rad, Marnes-la-Coquette, France). The cell culture medium was replaced on day 3 by a fresh medium (containing SEP or not, depending on the case), allowing for the continuation of the experiment until day 6.

#### 4.6.3. Extracellular Elastin Quantification—ELISA Assay

As previously described [28], VSMCs were cultured in 96-well plates in fresh 1% FBS-DMEM until they reached total confluency. They were then bathed in 1% FBS-DMEM supplemented with 0, 5, 10 or 50 µg/mL SEP. After 1, 3 or 6 days, the wells were washed three times with 0.1% Tween-containing PBS 1X, and VSMCs were exposed to the primary antibody to elastin (1:1500; 100 µL/well, 1 h, 37 °C) (ab21610, Abcam, Paris, France) before application of the secondary anti-rabbit antibody (1/5000, 100 µL/well, 1 h, 37 °C) (cat. #111-035-003, Jackson ImmunoResearch, West Grove, PA, USA) coupled to horseradish peroxidase (HRP). After triple washing with 0.1% Tween-containing PBS 1X, this was followed by addition of 100 µL/well substrate of HRP, 3,3′,5,5′-tetramethybenzidine (TMB, cat#T0440, Sigma-Aldrich, Saint-Louis, MO, USA) for 30 min at room temperature, the reaction being stopped by the addition of sulfuric acid (1M). After 10 min, the reaction end product was then quantified by measuring its absorbance at 450 nm with a multi-well spectrophotometer (1681000, Bio-Rad, Marnes-la-Coquette, France).

### 4.7. Statistical Analysis

Regarding the experiments other than aorta ring mechanics, reactivity and length, the statistical comparisons were performed by using two-, three- or four-way ANOVAs, followed, where necessary, by Fisher’s least significant difference (LSD) tests for paired value comparisons. The software used were Statistica 6.1 (StatSoft, Tulsa, OK, USA) and GraphPad Prism 4 (GraphPad Software. Inc., San Diego, CA, USA).

For aortic ring mechanics/reactivity, the concentration–response curves were fitted to a sigmoidal logistic equation using GraphPad Prism 4 (GraphPad Software. Inc., San Diego, CA, USA). The concentration–response curves and internal circumference–pressure curves were analyzed using two-way ANOVAs, followed by multiple Bonferroni post-tests. Unpaired t-tests were also used for comparison of the ascending aorta lengths.

The results are presented as mean values ± SEM, and *p*-values ≤ 0.05 were considered statistically significant.

## Figures and Tables

**Table 1:** Body weight, hematocrit and ratios of total heart and left ventricle plus septum weights to body weight.

	Male				Female			
	*Eln* *^+/+^*		*Eln* *^+/-^*		*Eln* *^+/+^*		*Eln* *^+/-^*	
	NaCl	SEP	NaCl	SEP	NaCl	SEP	NaCl	SEP
BW (g)	34.3 ± 1.2	34.7 ± 1.1	36.2 ± 1.6	34.1 ± 1.0	26.6 ± 0.8	25.7 ± 0.7	27.5 ± 1.2	27.3 ± 1.5
Hematocrit (%) ^&^	37.8 ± 1.4	38.7 ± 0.8	39.4 ± 1.3	40.7 ± 1.4	39.9 ± 1.0	37.9 ± 1.8	40.0 ± 1.8	35.7 ± 1.3
HW/BW (%) ^$^	0.46 ± 0.01	0.48 ± 0.01	0.48 ± 0.02	0.52 ± 0.01	0.47 ± 0.02	0.45 ± 0.01	0.49 ± 0.02	0.47 ± 0.02
LV+S/BW (%) ^$^	0.35 ± 0.01	0.37 ± 0.01	0.36 ± 0.02	0.41 ± 0.01	0.35 ± 0.01	0.32 ± 0.01	0.37± 0.01	0.35 ± 0.01

BW: Body weight, HW: Heart weight, LV: Left ventricle weight, S: Septum weight. ^&^ significant interaction between sex and treatment, independently of genotype (three-way ANOVA, *p* ≤ 0.05), showing that SEP decreased hematocrit in females, not males (post hoc Fisher’s least significant difference (LSD) tests, *p* ≤ 0.05). ^$^ significant interaction between sex and treatment, independently of genotype (three-way ANOVA, *p* ≤ 0.05), showing that, although no significant difference could be detected between untreated male and female mice, SEP treatment led to heavier HW/BW and LV+S/BW in male compared to female animals (post hoc Fisher’s least significant difference (LSD) tests, *p* ≤ 0.05). Control animals were injected with the same volume of 0.9% NaCl. Values are mean ± SEM. n = 7–10 per group.

## Data Availability

Data are not publicly archived. Data analyzed and generated during the study are available from the corresponding author G.F. on reasonable request.

## Supplementary Materials

The following supporting information can be downloaded at: https://www.mdpi.com/article/10.3390/ijms232113464/s1.

## Appendix A

### Appendix A.1

#### Detailed Surgical Procedure and Methods for Mechanical Study of Cannulated Aortae

Animals were anaesthetized by intraperitoneal injection of a ketamine (100 mg/kg)/xylazine (10 mg/kg) solution, and an injection of heparin (90 units/30 g) was performed in the saphenous vein. The ascending aorta was quickly excised and placed in a physiological buffer (135 mM NaCl, 5 mM KCl, 1.17 mM MgSO_4_, 0.44 mM KH_2_PO_4_, 2.6 mM NaHCO_3_, 0.34 mM Na_2_HPO_4_, 1.6 mM CaCl_2_-2H_2_O, 5.5 mM D-glucose, 0.025 mM EDTA, 10 mM HEPES; pH = 7.4). The vessels were cleaned of the adhering connective tissue and fat, then cannulated and mounted onto a pressure arteriograph, as described previously [12,58]. The pressure arteriograph was composed of a plexiglass organ bath (filled with 10 mL physiological buffer), surrounded by a water jacket for heat regulation, in which two aligned cannulae (diameter: ~1 mm) were supported by holders. Each cannula was ligatured to one end of the ascending aorta, while the opposite end of the cannula was connected to a small tube joining a reservoir filled with physiological buffer, also allowing for changes of the pressure in the tubing and vessel. A verification of the absence of physiological buffer leakage (because of the wrong ligation or presence of a small hole in the vessel) upon pressurization of the vessel was then performed prior to starting the experiments. The experiments were performed at 37 °C, and the physiological buffer present in the bath and the vessel was replaced every 15 min. Following a 30 min equilibration period, the vessel was transilluminated under an inverted microscope connected to a charge-coupled device camera and a computer. The proprietary software Windiam allowed for the continuous detection of the vessel’s inner and outer borders on the images from the camera (2 images/s) and recording of the vessel’s diameters [12]. Recordings of vessel inner diameters (IDs) and outer diameters (ODs) were taken while increasing the intravascular (transmural) pressure from 0 to 175 mmHg by steps of 25 mmHg (5 min per step). The inner diameter was directly measurable by transillumination at and above 125 mmHg because of the thinning of the vessel wall due to increasing pressure-induced dilatation, allowing a clear and contrasted image of the inner wall edge. At lower pressures, as previously described, the inner diameter can be accurately calculated through the entire range of pressures tested from the measured OD and a unique measurement of the vessel wall cross-sectional area (WCSA). As previously described [19,58], assuming that the WCSA is constant, the formula finally used to calculate ID was

ID_x mmHg_ = √(OD^2^_x mmHg_ − OD^2^_175 mmHg_ + ID^2^_175 mmHg_) (A1)

Distensibility is defined as a change in the relative volume (percentage) of the lumen per pressure unit during a change in the intravascular pressure, as described by Smith and Kampine [68]. Distensibility (D), in percent, is

D = 100(∆V/VA)/∆P (A2)

V_A_ is the luminal initial volume before a small change in pressure. ∆V is the luminal volume change (V_A_ to V_B_) in response to a change in transmural pressure (from pressure A to B). The luminal volume of a given constant length (L) of a vessel segment is L.π.ID^2^/4. Therefore

D = 100 ([L π ∆(ID^2^_B_/4)]/[L π ID^2^_A_/4])/∆P (A3)

D = 100(∆(ID^2^_B_)/ID^2^_A_)/∆P = 100 [(ID^2^_B_–ID^2^_A_)/ID^2^_A_]/∆P (A4)

Since the pressure change increment in our experiments was 25 mmHg, we replaced, in the following sections, the distensibility (% change in volume per 1 mmHg) by distensibility per 25 mmHg increment (D25). In any pressure range, a 25 mmHg pressure change between pressure X mmHg and pressure X + 25 mmHg leads to the following expression of D25.

D25 = 100(ID^2^_X + 25_ – ID^2^_X_)/ID^2^_X_ (A5)

The circumferential midwall strain and circumferential wall stress were calculated according to the formulae given by Gibbons and Shadwick [59]:

Circumferential midwall strain (ε)

ε = ∆R/R0 = (R − R0)/R0 (A6)

where R is the midwall radius (R = (OD + ID)/4), and R0 is the midwall radius at no transmural pressure (0 mmHg).

Circumferential wall stress (σ)

σ = (P × r)/h (A7)

where P is the pressure (in kPa), r is the luminal radius (= ID/2), and h is the wall thickness.

σ represents the forces that are circumferentially applied to each small portion (surface) of the vessel wall because of the intraluminal pressure-induced stretching of the vessel.

The incremental elastic modulus (Einc, 25 mmHg increments) represents the stiffness of the arterial wall material:

Einc = 0.75 (1 + ε) ∆σ/∆ε (A8)

1 kPa is assumed to be equal to 7.5006 mmHg.

### Appendix A.2

The primers used in RT-qPCR studies were as follows:

**Table:**

mRNA	Forward Primer (5′−3′)	Reverse Primer (5′−3′)
**collagen type I, alpha 1 (*Col1a1*)**	GCGAAGGCAACAGTCGATTC	CCCAAGTTCCGGTGTGACTC
**collagen type I, alpha 2 (*Col1a2*)**	CAAGCATGTCTGGTTAGGAGAG	AGGACACCCCTTCTACGTTGT
**collagen type III, alpha 1 (*Col3a1*)**	CAGCTGGCCTTCCTCAGACTT	GCTGTTTTTGCAGTGGTATGTAATG
**fibrillin1 (*Fbn1*)**	TGCCAGCAGCGAGATGGACGA	TGGCGAGGCTCACGTTGGCTT
**fibulin 5 (*Fbln5*)**	TTGAGGAAGATGGCATTCACT	GGCTGGTTCACACACTCGT
**lysyl oxidase (*Lox*)**	AATTCAGCCACTATGACCTGCTTGA	GTAGCGAATGTCACAGCGTACAACA
**lysyl oxidase-like-1 (*Loxl1*)**	TATGCCTGCACCTCTCACAC	TGTCCGCATTGTATGTGTCAT
**tropoelastin (*Eln*)**	AAGCTGCTGCTAAGGCTGC	TGCAACTCCTCCACCTGGGAA
A: adenine, C: cytosine, G: guanine, T: thymine.		

## References

[1] Tucker W.D., Mahajan K. (2019). Anatomy, blood vessels. StatPearls.

[2] Greenwald S.E. (2007). Ageing of the Conduit Arteries. J. Pathol..

[3] O’Rourke M.F., Hashimoto J. (2007). Mechanical Factors in Arterial Aging: A Clinical Perspective. J. Am. Coll. Cardiol..

[4] Chapter 7: The Cardiovascular System: Blood Vessels and Circulation—Anatomy & Physiology. https://qut.pressbooks.pub/anatomyandphysiology/chapter/chapter-20-the-cardiovascular-system-blood-vessels-and-circulation/.

[5] Behmoaras J., Osborne-Pellegrin M., Gauguier D., Jacob M.-P. (2005). Characteristics of the Aortic Elastic Network and Related Phenotypes in Seven Inbred Rat Strains. Am. J. Physiol. Heart Circ. Physiol..

[6] Leung D.Y., Glagov S., Mathews M.B. (1977). Elastin and Collagen Accumulation in Rabbit Ascending Aorta and Pulmonary Trunk during Postnatal Growth. Correlation of Cellular Synthetic Response with Medial Tension. Circ. Res..

[7] Wolinsky H., Glagov S. (1964). Structural Basis for the Static Mechanical Properties of the Aortic Media. Circ. Res..

[8] Wolinsky H., Glagov S. (1967). A Lamellar Unit of Aortic Medial Structure and Function in Mammals. Circ. Res..

[9] Fhayli W., Boëté Q., Harki O., Briançon-Marjollet A., Jacob M.-P., Faury G. (2019). Rise and Fall of Elastic Fibers from Development to Aging. Consequences on Arterial Structure-Function and Therapeutical Perspectives. Matrix Biol..

[10] Baldwin A.K., Simpson A., Steer R., Cain S.A., Kielty C.M. (2013). Elastic Fibres in Health and Disease. Expert Rev. Mol. Med..

[11] Arnaud C., Beguin P., Lantuejoul S., Pepin J.-L., Guillermet C., Pelli G., Burger F., Buatois V., Ribuot C., Baguet J.-P. (2011). The Inflammatory Preatherosclerotic Remodeling Induced by Intermittent Hypoxia Is Attenuated by RANTES/CCL5 Inhibition. Am. J. Respir. Crit. Care Med..

[12] Pezet M., Jacob M.-P., Escoubet B., Gheduzzi D., Tillet E., Perret P., Huber P., Quaglino D., Vranckx R., Li D.Y. (2008). Elastin Haploinsufficiency Induces Alternative Aging Processes in the Aorta. Rejuvenat. Res..

[13] Mariko B., Pezet M., Escoubet B., Bouillot S., Andrieu J.-P., Starcher B., Quaglino D., Jacob M.-P., Huber P., Ramirez F. (2011). Fibrillin-1 Genetic Deficiency Leads to Pathological Ageing of Arteries in Mice. J. Pathol..

[14] Duque Lasio M.L., Kozel B.A. (2018). Elastin-Driven Genetic Diseases. Matrix Biol..

[15] Kassai B., Bouyé P., Gilbert-Dussardier B., Godart F., Thambo J.-B., Rossi M., Cochat P., Chirossel P., Luong S., Serusclat A. (2019). Minoxidil versus Placebo in the Treatment of Arterial Wall Hypertrophy in Children with Williams Beuren Syndrome: A Randomized Controlled Trial. BMC Pediatr..

[16] Curran M.E., Atkinson D.L., Ewart A.K., Morris C.A., Leppert M.F., Keating M.T. (1993). The Elastin Gene Is Disrupted by a Translocation Associated with Supravalvular Aortic Stenosis. Cell.

[17] Ewart A.K., Morris C.A., Atkinson D., Jin W., Sternes K., Spallone P., Stock A.D., Leppert M., Keating M.T. (1993). Hemizygosity at the Elastin Locus in a Developmental Disorder, Williams Syndrome. Nat. Genet..

[18] Li D.Y., Faury G., Taylor D.G., Davis E.C., Boyle W.A., Mecham R.P., Stenzel P., Boak B., Keating M.T. (1998). Novel Arterial Pathology in Mice and Humans Hemizygous for Elastin. J. Clin. Investig..

[19] Faury G., Pezet M., Knutsen R.H., Boyle W.A., Heximer S.P., McLean S.E., Minkes R.K., Blumer K.J., Kovacs A., Kelly D.P. (2003). Developmental Adaptation of the Mouse Cardiovascular System to Elastin Haploinsufficiency. J. Clin. Investig..

[20] Sproul E.P., Argraves W.S. (2013). A Cytokine Axis Regulates Elastin Formation and Degradation. Matrix Biol..

[21] Hayashi A., Suzuki T., Wachi H., Tajima S., Nishikawa T., Murad S., Pinnell S.R. (1994). Minoxidil Stimulates Elastin Expression in Aortic Smooth Muscle Cells. Arch. Biochem. Biophys..

[22] Tokimitsu I., Tajima S. (1994). Inhibition of Elastin Synthesis by High Potassium Salt Is Mediated by Ca2+ Influx in Cultured Smooth Muscle Cells in Vitro: Reciprocal Effects of K+ on Elastin and Collagen Synthesis. J. Biochem..

[23] Lannoy M., Slove S., Louedec L., Choqueux C., Journé C., Michel J.-B., Jacob M.-P. (2014). Inhibition of ERK1/2 Phosphorylation: A New Strategy to Stimulate Elastogenesis in the Aorta. Hypertension.

[24] Tsoporis J., Keeley F.W., Lee R.M., Leenen F.H. (1998). Arterial Vasodilation and Vascular Connective Tissue Changes in Spontaneously Hypertensive Rats. J. Cardiovasc. Pharmacol..

[25] Knutsen R.H., Beeman S.C., Broekelmann T.J., Liu D., Tsang K.M., Kovacs A., Ye L., Danback J.R., Watson A., Wardlaw A. (2018). Minoxidil Improves Vascular Compliance, Restores Cerebral Blood Flow, and Alters Extracellular Matrix Gene Expression in a Model of Chronic Vascular Stiffness. Am. J. Physiol. Heart Circ. Physiol..

[26] Slove S., Lannoy M., Behmoaras J., Pezet M., Sloboda N., Lacolley P., Escoubet B., Buján J., Jacob M.-P. (2013). Potassium Channel Openers Increase Aortic Elastic Fiber Formation and Reverse the Genetically Determined Elastin Deficit in the BN Rat. Hypertension.

[27] Coquand-Gandit M., Jacob M.-P., Fhayli W., Romero B., Georgieva M., Bouillot S., Estève E., Andrieu J.-P., Brasseur S., Bouyon S. (2017). Chronic Treatment with Minoxidil Induces Elastic Fiber Neosynthesis and Functional Improvement in the Aorta of Aged Mice. Rejuvenat. Res..

[28] Fhayli W., Boyer M., Ghandour Z., Jacob M.P., Andrieu J.P., Starcher B.C., Estève E., Faury G. (2019). Chronic Administration of Minoxidil Protects Elastic Fibers and Stimulates Their Neosynthesis with Improvement of the Aorta Mechanics in Mice. Cell Signal.

[29] Raveaud S., Mezin P., Lavanchy N., Starcher B., Mecham R.P., Verdetti J., Faury G. (2009). Effects of Chronic Treatment with a Low Dose of Nicorandil on the Function of the Rat Aorta during Ageing. Clin. Exp. Pharmacol. Physiol..

[30] Bouhedja M., Peres B., Fhayli W., Ghandour Z., Boumendjel A., Faury G., Khelili S. (2018). Design, Synthesis and Biological Evaluation of Novel Ring-Opened Cromakalim Analogues with Relaxant Effects on Vascular and Respiratory Smooth Muscles and as Stimulators of Elastin Synthesis. Eur. J. Med. Chem..

[31] Bouider N., Fhayli W., Ghandour Z., Boyer M., Harrouche K., Florence X., Pirotte B., Lebrun P., Faury G., Khelili S. (2015). Design and Synthesis of New Potassium Channel Activators Derived from the Ring Opening of Diazoxide: Study of Their Vasodilatory Effect, Stimulation of Elastin Synthesis and Inhibitory Effect on Insulin Release. Bioorg. Med. Chem..

[32] Fhayli W., Boëté Q., Kihal N., Cenizo V., Sommer P., Boyle W.A., Jacob M.-P., Faury G. (2020). Dill Extract Induces Elastic Fiber Neosynthesis and Functional Improvement in the Ascending Aorta of Aged Mice with Reversal of Age-Dependent Cardiac Hypertrophy and Involvement of Lysyl Oxidase-Like-1. Biomolecules.

[33] Nonaka R., Sato F., Wachi H. (2014). Domain 36 of Tropoelastin in Elastic Fiber Formation. Biol. Pharm. Bull..

[34] Bax D.V., Rodgers U.R., Bilek M.M.M., Weiss A.S. (2009). Cell Adhesion to Tropoelastin Is Mediated via the C-Terminal GRKRK Motif and Integrin AlphaVbeta3. J. Biol. Chem..

[35] Faury G. (1998). Role of the Elastin-Laminin Receptor in the Cardiovascular System. Pathol. Biol..

[36] Rodgers U.R., Weiss A.S. (2004). Integrin Alpha v Beta 3 Binds a Unique Non-RGD Site near the C-Terminus of Human Tropoelastin. Biochimie.

[37] Wahart A., Hocine T., Albrecht C., Henry A., Sarazin T., Martiny L., El Btaouri H., Maurice P., Bennasroune A., Romier-Crouzet B. (2019). Role of Elastin Peptides and Elastin Receptor Complex in Metabolic and Cardiovascular Diseases. FEBS J..

[38] Yamashiro Y., Yanagisawa H. (2020). The Molecular Mechanism of Mechanotransduction in Vascular Homeostasis and Disease. Clin. Sci..

[39] Chen J., Yu J., Yuan R., Li N., Li C., Zhang X. (2021). MTOR Inhibitor Improves Testosterone-Induced Myocardial Hypertrophy in Hypertensive Rats. J. Endocrinol..

[40] Robinet A., Millart H., Oszust F., Hornebeck W., Bellon G. (2007). Binding of Elastin Peptides to S-Gal Protects the Heart against Ischemia/Reperfusion Injury by Triggering the RISK Pathway. FASEB J..

[41] Azevedo J., Arroja I., Jacques A., Santos I., Amado P., Marques J.C., Araújo V. (1993). A double ambulatory product (blood pressure and heart rate), mild arterial hypertension and left ventricular hypertrophy. Rev. Port. Cardiol..

[42] Sahu S.P., Liu Q., Prasad A., Hasan S.M.A., Liu Q., Rodriguez M.X.B., Mukhopadhyay O., Burk D., Francis J., Mukhopadhyay S. (2020). Characterization of Fibrillar Collagen Isoforms in Infarcted Mouse Hearts Using Second Harmonic Generation Imaging. Biomed. Opt. Express.

[43] McNulty M., Mahmud A., Spiers P., Feely J. (2006). Collagen Type-I Degradation Is Related to Arterial Stiffness in Hypertensive and Normotensive Subjects. J. Hum. Hypertens..

[44] Li D.Y., Brooke B., Davis E.C., Mecham R.P., Sorensen L.K., Boak B.B., Eichwald E., Keating M.T. (1998). Elastin Is an Essential Determinant of Arterial Morphogenesis. Nature.

[45] Urbán Z., Riazi S., Seidl T.L., Katahira J., Smoot L.B., Chitayat D., Boyd C.D., Hinek A. (2002). Connection between Elastin Haploinsufficiency and Increased Cell Proliferation in Patients with Supravalvular Aortic Stenosis and Williams-Beuren Syndrome. Am. J. Hum. Genet..

[46] Karnik S.K., Brooke B.S., Bayes-Genis A., Sorensen L., Wythe J.D., Schwartz R.S., Keating M.T., Li D.Y. (2003). A Critical Role for Elastin Signaling in Vascular Morphogenesis and Disease. Development.

[47] Li W., Li Q., Qin L., Ali R., Qyang Y., Tassabehji M., Pober B.R., Sessa W.C., Giordano F.J., Tellides G. (2013). Rapamycin Inhibits Smooth Muscle Cell Proliferation and Obstructive Arteriopathy Attributable to Elastin Deficiency. Arterioscler. Thromb. Vasc. Biol..

[48] Wachi H., Seyama Y., Yamashita S., Suganami H., Uemura Y., Okamoto K., Yamada H., Tajima S. (1995). Stimulation of Cell Proliferation and Autoregulation of Elastin Expression by Elastin Peptide VPGVG in Cultured Chick Vascular Smooth Muscle Cells. FEBS Lett..

[49] Debret R., Faye C., Sohier J., Sommer P. (2017). Polypeptide Derive de la Tropoelastine et Materiau Biocompatible le Comprenant. WIPO Patent.

[50] Le Page A., Khalil A., Vermette P., Frost E.H., Larbi A., Witkowski J.M., Fulop T. (2019). The Role of Elastin-Derived Peptides in Human Physiology and Diseases. Matrix Biol..

[51] Karnik S.K., Wythe J.D., Sorensen L., Brooke B.S., Urness L.D., Li D.Y. (2003). Elastin Induces Myofibrillogenesis via a Specific Domain, VGVAPG. Matrix Biol..

[52] Owens E.A., Jie L., Reyes B.A.S., Van Bockstaele E.J., Osei-Owusu P. (2017). Elastin Insufficiency Causes Hypertension, Structural Defects and Abnormal Remodeling of Renal Vascular Signaling. Kidney Int..

[53] Faury G., Ristori M.T., Verdetti J., Jacob M.P., Robert L. (1994). Role of the elastin-laminin receptor in the vasoregulation. C. R. Acad. Sci. III.

[54] Faury G., Ristori M.T., Verdetti J., Jacob M.P., Robert L. (1995). Effect of Elastin Peptides on Vascular Tone. J. Vasc. Res..

[55] Faury G., Chabaud A., Ristori M.T., Robert L., Verdetti J. (1997). Effect of Age on the Vasodilatory Action of Elastin Peptides. Mech. Ageing Dev..

[56] Faury G., Garnier S., Weiss A.S., Wallach J., Fülöp T., Jacob M.P., Mecham R.P., Robert L., Verdetti J. (1998). Action of Tropoelastin and Synthetic Elastin Sequences on Vascular Tone and on Free Ca2+ Level in Human Vascular Endothelial Cells. Circ. Res..

[57] Meyer D.E., Chilkoti A. (1999). Purification of Recombinant Proteins by Fusion with Thermally-Responsive Polypeptides. Nat. Biotechnol..

[58] Faury G., Maher G.M., Li D.Y., Keating M.T., Mecham R.P., Boyle W.A. (1999). Relation between Outer and Luminal Diameter in Cannulated Arteries. Am. J. Physiol..

[59] Gibbons C.A., Shadwick R.E. (1989). Functional Similarities in the Mechanical Design of the Aorta in Lower Vertebrates and Mammals. Experientia.

[60] Liu K.L., Canaple L., Del Carmine P., Gauthier K., Beylot M., Lo M. (2016). Thyroid Hormone Receptor-α Deletion Decreases Heart Function and Exercise Performance in Apolipoprotein E-Deficient Mice. Physiol. Genom..

[61] Shikata T., Uzawa T., Yoshiwara N., Akatsuka T., Yamazaki S. (1974). Staining Methods of Australia Antigen in Paraffin Section—Detection of Cytoplasmic Inclusion Bodies. Jpn. J. Exp. Med..

[62] Henwood T. (1983). Shikata’s Orcein Stain—A Routine Stain for Liver Biopsies. Aust. J. Med. Lab. Sci..

[63] Bauman T.M., Nicholson T.M., Abler L.L., Eliceiri K.W., Huang W., Vezina C.M., Ricke W.A. (2014). Characterization of Fibrillar Collagens and Extracellular Matrix of Glandular Benign Prostatic Hyperplasia Nodules. PLoS ONE.

[64] Bredfeldt J.S., Liu Y., Pehlke C.A., Conklin M.W., Szulczewski J.M., Inman D.R., Keely P.J., Nowak R.D., Mackie T.R., Eliceiri K.W. (2014). Computational Segmentation of Collagen Fibers from Second-Harmonic Generation Images of Breast Cancer. J. Biomed. Opt..

[65] Liu Y., Keikhosravi A., Mehta G.S., Drifka C.R., Eliceiri K.W. (2017). Methods for Quantifying Fibrillar Collagen Alignment. Methods Mol. Biol..

[66] Liu Y., Keikhosravi A., Pehlke C.A., Bredfeldt J.S., Dutson M., Liu H., Mehta G.S., Claus R., Patel A.J., Conklin M.W. (2020). Fibrillar Collagen Quantification With Curvelet Transform Based Computational Methods. Front. Bioeng. Biotechnol..

[67] CT-FIRE https://eliceirilab.org/software/ctfire/.

[68] Smith J.J., Kampine J.P. (1990). Circulatory Physiology: The Essentials.

