# Supplementary material for: Physiological Impact of a Synthetic Elastic Protein in Arterial Diseases Related to Alterations of Elastic Fibers: Effect on the Aorta of Elastin-Haploinsufficient Male and Female Mice
Source: Int J Mol Sci. 2022 Nov 3;23(21):13464. doi: 10.3390/ijms232113464 (PMC9656458; doi:10.3390/ijms232113464)
Supplement: Supplementary file 1 [file ijms-23-13464-s001.zip › ijms-1941375-supplementary.pdf]

Supplementary data

Supplementary data S1. Effects of sex and genotype on the measured parameters, independently of SEP treatment

1. Body and heart weights, hematocrit

Hematocrit was similar in the 8 groups. Body weight and left ventricle weight were generally found lower in female than male mice, independently of SEP treatment or genotype. Also, confirming our previous results [12,19], a general significant total heart and left ventricle hypertrophy was generally observed in $Eln^{+/-}$ mice, compared to their $Eln^{+/+}$ counterparts, independently of sex and SEP treatment (Supplementary data S1.1).

Supplementary data S1.1. Body weight, hematocrit and ratios of total heart and left ventricle plus septum weights to body weight.

	**Male**				**Female**			
	Eln $^{+/+}$		***Eln*** $^{+/-}$		***Eln*** $^{+/+}$		***Eln*** $^{+/-}$	
	**NaCl**	**SEP**	**NaCl**	**SEP**	**NaCl**	**SEP**	**NaCl**	**SEP**
**BW (g)** $^{£}$	34.3 ± 1.2	34.7 ± 1.1	36.2 ± 1.6	34.1 ± 1.0	26.6 ± 0.8	25.7 ± 0.7	27.5 ± 1.2	27.3 ± 1.5
**Hematocrit (%)**	37.8 ± 1.4	38.7 ± 0.8	39.4 ± 1.3	40.7 ± 1.4	39.9 ± 1.0	37.9 ± 1.8	40.0 ± 1.8	35.7 ± 1.3
**HW/BW (%)** *	0.46 ± 0.01	0.48 ± 0.01	0.48 ± 0.02	0.52 ± 0.01	0.47 ± 0.02	0.45 ± 0.01	0.49 ± 0.02	0.47 ± 0.02
**LV+S/BW (%)** $^{*\ £}$	0.35 ± 0.01	0.37 ± 0.01	0.36 ± 0.02	0.41 ± 0.01	0.35 ± 0.01	0.32 ± 0.01	0.37± 0.01	0.35 ± 0.01

BW: body weight, HW: heart weight, LV: left ventricle weight, S: septum weight. $^{£}$ general significant difference between male and female mice, independently of SEP treatment and genotype (three-way ANOVA, p≤0.05). *general significant difference between $Eln^{+/+}$ and $Eln^{+/-}$ mice, independently of SEP treatment and sex (three-way ANOVA, p≤0.05). Control animals were injected with the same volume of 0.9% NaCl. Values are mean ± SEM. n = 7-10 per group.

2.2. Blood pressure

As expected [12,19], blood pressures, measured by carotid artery catheterization, were substantially increased in adult $Eln^{+/-}$ (SBP = 133 ± 6 mmHg and 140 ± 5 mmHg in females and males, respectively) compared to $Eln^{+/+}$ animals (SBP = 113 ± 6 mmHg and 118 ± 9 mmHg in females and males, respectively), independently of treatment and sex (four-way ANOVA, p≤0.05) (Supplementary data S1.2).

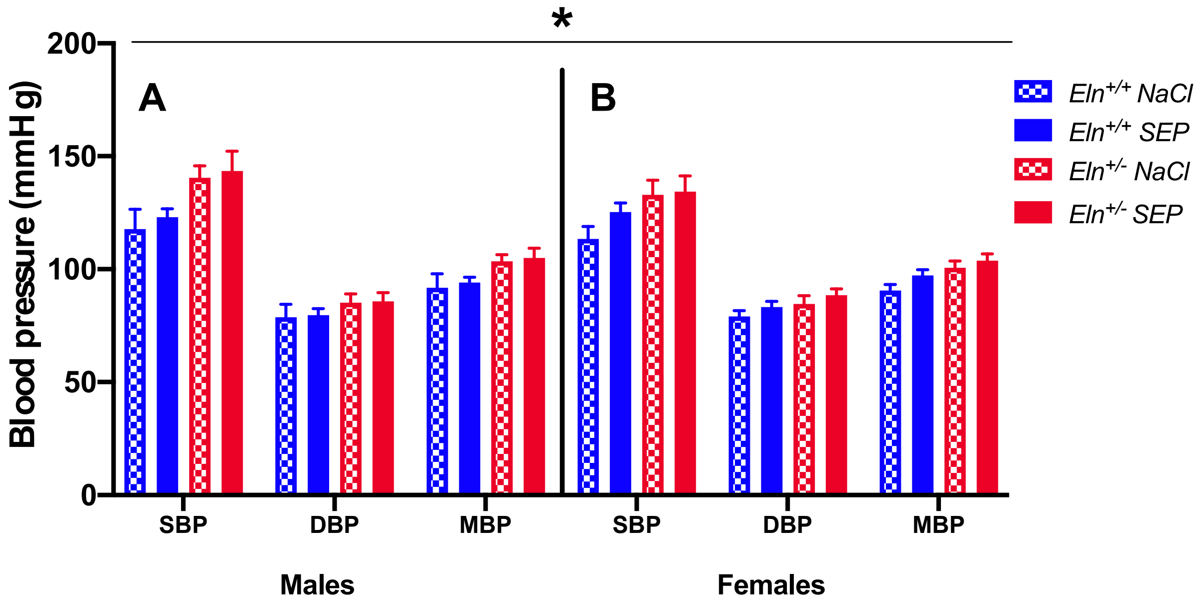

Supplementary data S1.2. Systolic, mean and diastolic arterial blood pressures. The measurements were performed 3 days after NaCl (control) or SEP intravenous injection in the caudal vein of male and female mice of *Eln*$^{+/+}$ or *Eln*$^{+/-}$ genotypes. (A) Males, (B) Females, SBP: Systolic Blood Pressure, DBP: Diastolic Blood Pressure, MBP: Mean Blood Pressure. *general significant difference between *Eln*$^{+/+}$ or *Eln*$^{+/-}$ mice, independently of sex and SEP treatment (four-way ANOVA, p≤0.05). Control animals were injected with the same volume of 0.9% NaCl. Values are mean ± SEM. n = 4–7 per group.

2.3. Biomechanics of the cannulated ascending aorta

Ex-vivo biomechanical studies of the cannulated ascending aorta showed that outer (OD) and inner diameters (ID) were generally larger in males than females, independently of genotype and SEP treatment (four-way ANOVA, p≤0.05). Also, independently of pressure, it was observed a general decrease in: i) arterial ID and OD in Eln$^{+/-}$ mice of both sexes, and ii) wall thickness in male *Eln*$^{+/-}$ mice only, compared to corresponding *Eln*$^{+/+}$ animals (Supplementary data S1.3).

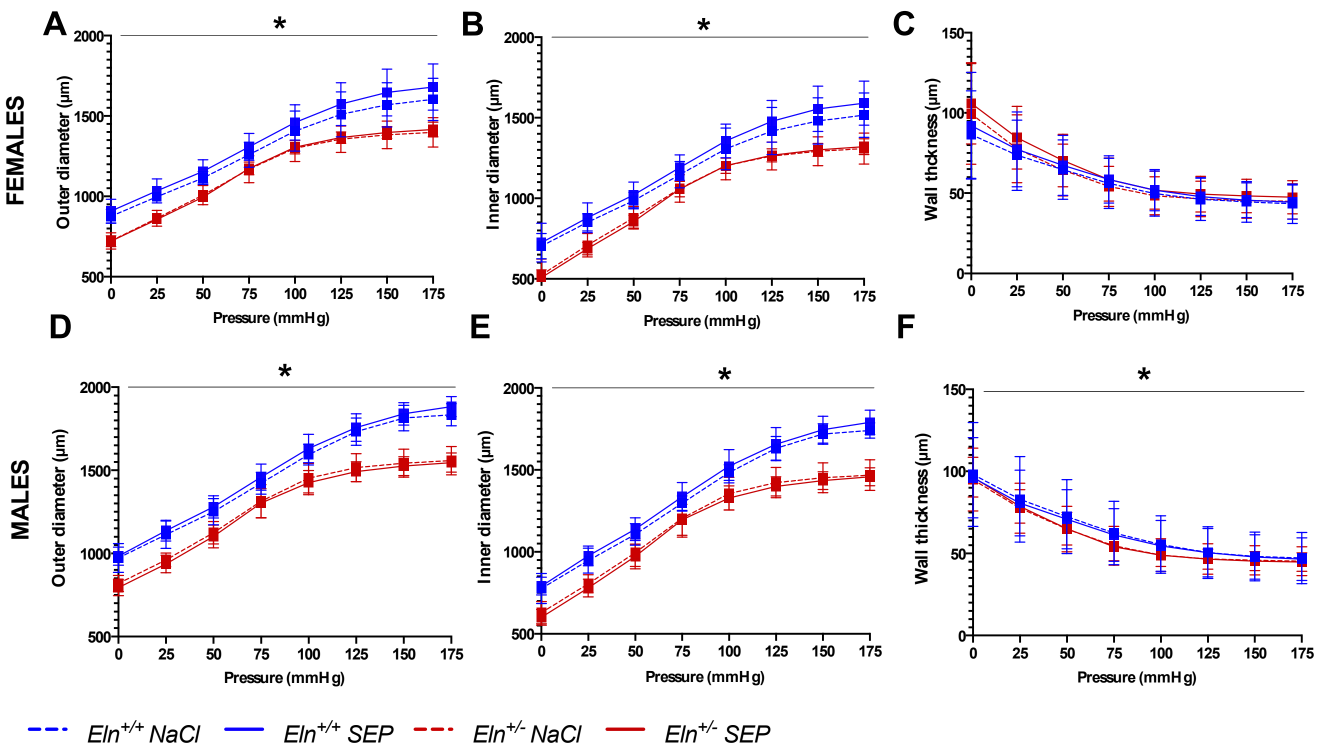

Supplementary data S1.3. Diameter-pressure and wall-thickness-pressure curves of the cannulated ascending aorta. The measurements were performed by pressure arteriography, 3 days after NaCl (control) or SEP intravenous injection in the caudal vein of male and female mice of *Eln*$^{+/+}$ or *Eln*$^{+/-}$ genotypes (A, B, C: females ; D, E, F: males). (A,D): outer diameter-pressure relation. (B,E): inner diameter-pressure relation. (C,F): wall thickness-pressure relation. *general significant difference between *Eln*$^{+/+}$ or *Eln*$^{+/-}$ mice of each sex, independently of SEP treatment (three-way ANOVA, $p \leq 0.05$). Control animals were injected with the same volume of 0.9% NaCl. Values are mean ± SEM. n = 7–10 per group.

Genotype affected all the studied mechanical parameters, i.e. distensibility, wall stress, wall strain and incremental elastic modulus, in animals of both sexes and independently of intraluminal pressure (Supplementary data S1.4). In *Eln*$^{+/-}$ animals, distensibility (D25) was increased at low intraluminal pressure and decreased at the highest pressures compared to that of *Eln*$^{+/+}$ mice (Supplementary data S1.4A,E). Circumferential wall stress, inversely proportional to wall thickness, was generally found lower (Supplementary data S1.4B,F) and circumferential wall strain was found higher in *Eln*$^{+/-}$ animals of both sexes, compared to wild type mice (Supplementary data S1.4C,G). Incremental elastic modulus (Einc), indicative of the wall material stiffness, was generally and significantly increased in *Eln*$^{+/-}$ mice of both sexes as expected, and was particularly marked in the 125-175 mmHg range (Supplementary data S1.4D,H). No general effect of sex could be detected regarding these parameters (D25, wall stress, wall strain and Einc) (Supplementary data S1.4).

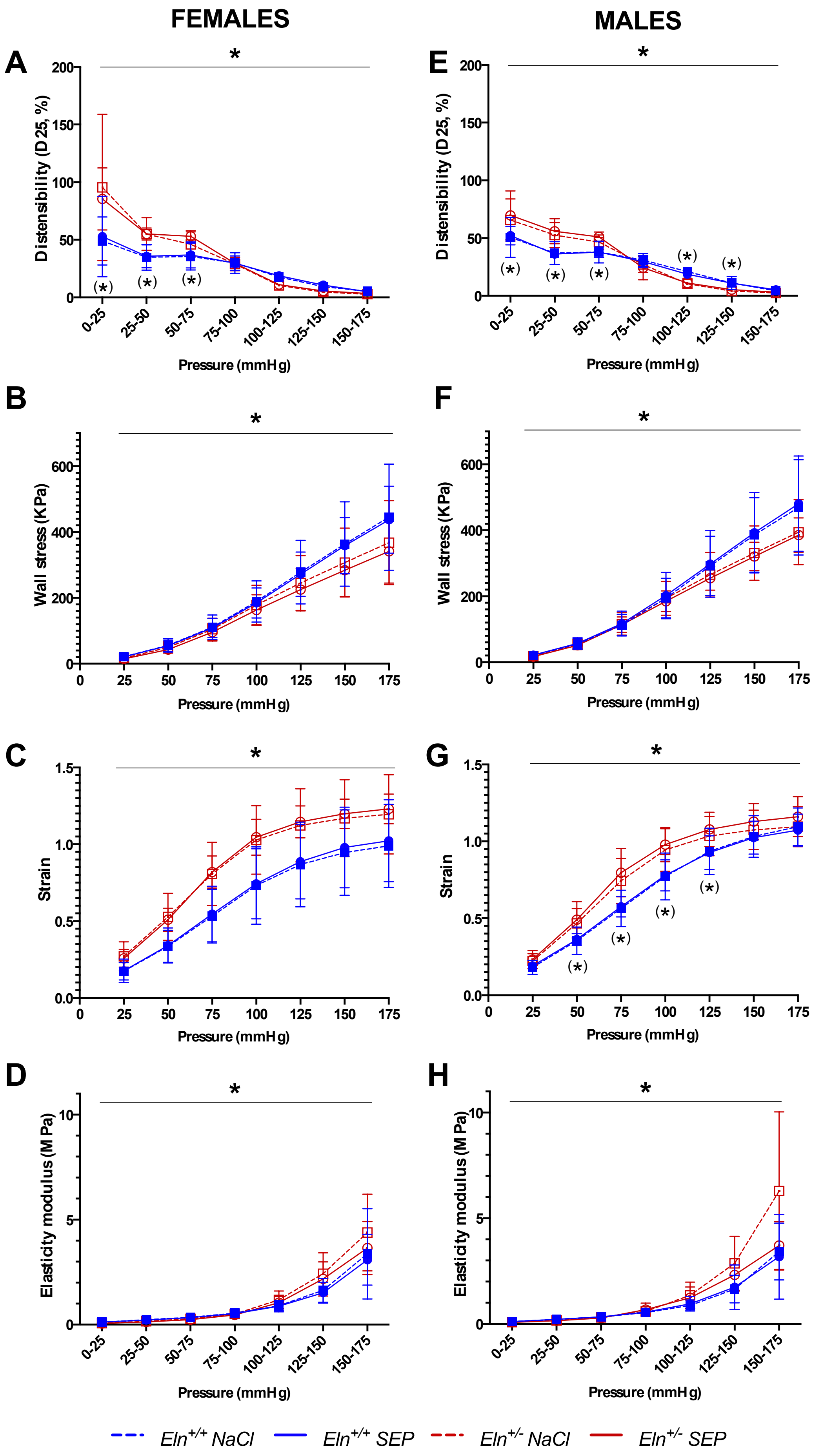

FEMALES
MALES
A
B
C
D
E
F
G
H
Distensibility (D25, %)
Wall stress (KPa)
Strain
Elasticity modulus (MPa)
Pressure (mmHg)
0-25
25-50
50-75
75-100
100-125
125-150
150-175
Eln+/+ NaCl
Eln+/+ SEP
Eln+/- NaCl
Eln+/- SEP

Supplementary data S1.4. Mechanical parameters of the cannulated ascending aorta. The measurements were performed by pressure arteriography 3 days after NaCl (control) or SEP intravenous injection in the caudal vein of male and female mice of *Eln*$^{+/+}$ or *Eln*$^{+/-}$ genotypes. (A,E) aortic distensibility per 25 mmHg increment (D25)-pressure relation; (B,F) circumferential stress-pressure relation; (C,G) circumferential strain-pressure relation; (D,H) incremental elastic modulus (Einc)-pressure increment relation. *general significant difference between *Eln*$^{+/+}$ and *Eln*$^{+/-}$ mice, independently of SEP treatment (three-way ANOVA, p≤0.05). (*) significant difference at a specific pressure between *Eln*$^{+/+}$ and *Eln*$^{+/-}$ mice, independently of SEP treatment (three-way ANOVA showing a significant interaction between genotype and pressure, followed by post hoc Fishers's Least Significant Difference (LSD) test for paired comparisons, p≤0.05). Control animals were injected with the same volume of 0.9% NaCl. Values are mean ± SEM. n = 7–10 per group.

2.4. Ascending aorta ring reactivity and mechanics

Tension arteriography showed that the vasoconstriction of ascending aorta rings induced by acute application of phenylephrine (Phe) did not differ between *Eln*$^{+/-}$ and *Eln*$^{+/+}$ mice of both sexes, while acetylcholine (Ach)-induced relaxation was of significantly lower amplitude than that of the other three groups in female *Eln*$^{+/-}$ mice only. This confirms our previous results obtained in 6-month-old *Eln*$^{+/-}$ and *Eln*$^{+/+}$ male mice [12,19]. No inter-group difference was observed for acute sodium nitroprusside (SNP)-induced relaxation (Supplementary data S1.5, three left panels for each sex).

The ascending aorta circumference-transmural pressure relation, indicative of arterial stiffness, was then studied. The internal circumference determined at an effective tension of 90 mmHg was significantly lower ($p < 0.001$) in *Eln*$^{+/-}$ (3.13 ± 0.08 and 3.53 ± 0.08 mm for female and male mice, respectively) than *Eln*$^{+/+}$ mice (4.04 ± 0.16 and 4.22 ± 0.12 mm for female and male mice, respectively). In response to increasing stretches from the internal circumference of 2.6 mm, *Eln*$^{+/-}$ mice had greater transmural pressure than *Eln*$^{+/+}$ mice for both sexes, indicative of stiffer aortae in *Eln*$^{+/-}$ animals (Supplementary data S1.5, right panels).

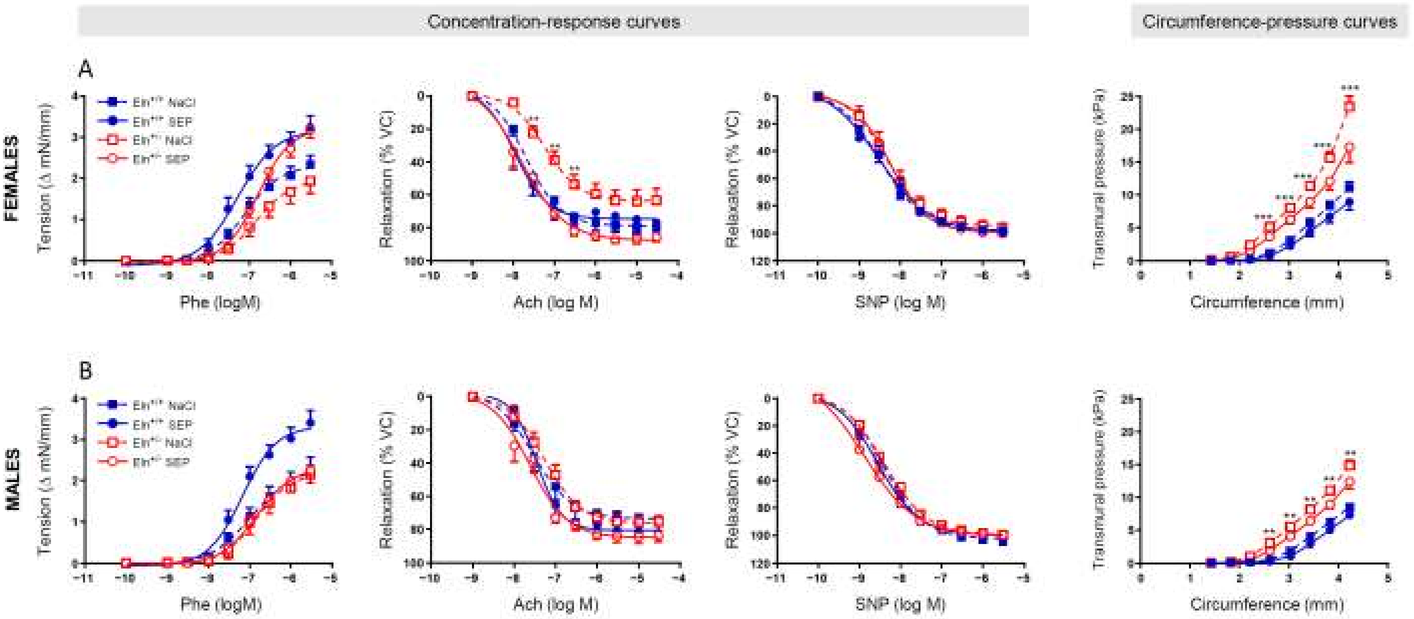

Supplementary data S1.5. Concentration-response and circumference-pressure curves of the ascending aorta. The studies were performed by wire myography in aortae from female (A) and male (B) *Eln*$^{+/+}$ and *Eln*$^{+/-}$ mice treated with single 0.9% NaCl (NaCl, controls) or synthetic elastic protein (SEP) intravenous injection three days before the experiments. ** p<0.01 and *** p<0.001 *Eln*$^{+/-}$NaCl vs *Eln*$^{+/+}$NaCl (two-way ANOVAs followed, when necessary, by multiple Bonferroni post-tests). Values are mean ± SEM. n=11-14 in each C group; n=7-8 in each SEP group.

2.5. Aorta morphology and structure

First, the ascending aorta was significantly longer in *Eln*$^{+/-}$ (2.57 ± 0.06 and 2.65 ± 0.08 mm in females and males, respectively) than in *Eln*$^{+/+}$ mice (2.10 ± 0.07 and 2.01 ± 0.08 mm in females and males, respectively) (unpaired t-test, P≤0.05). This confirms our previous results obtained in male mice [12] and extends this finding to female animals. Histological analysis of orcein-stained aortae (see Figure 5 of the main article for representative images) indicated that no significant difference between groups could be observed regarding the media and interlamellar thicknesses (Supplementary data S1.6A,D). However, elastic lamellae were more numerous although thinner (except for lamella #5) in *Eln*$^{+/-}$ mice compared to their *Eln*$^{+/+}$ counterparts, independently of sex and treatment (Supplementary data S1.6B,C), in accordance with the literature [18]. The number of elastic lamella disruptions was generally higher in *Eln*$^{+/-}$ than in *Eln*$^{+/+}$ animals, in accordance with our previous results [12] (Supplementary data S1.6E). No sex-related difference could be detected.

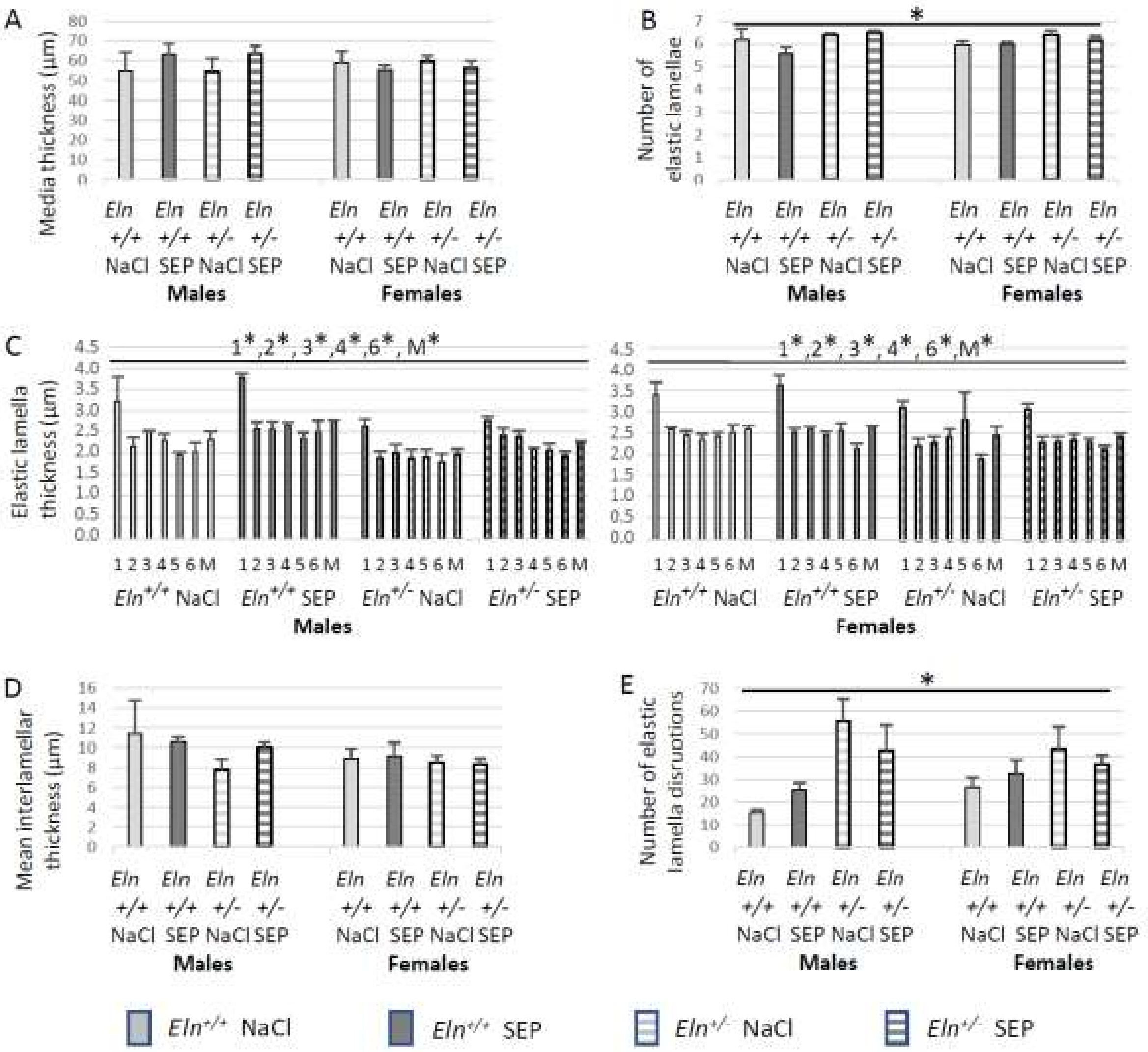

Supplementary data S1.6. Morphology and structure of the distal part of the aortic arch analyzed by orcein staining depending on genotype, sex and SEP treatment. The measurements were performed three days after NaCl (control) or SEP intravenous injection in the caudal vein of male and female mice of *Eln*$^{+/+}$ or *Eln*$^{+/-}$ genotypes. (A) Media thickness. (B) Number of elastic lamellae. (C) Mean thickness of the elastic lamellae (M) and thickness of each elastic lamellae, numbered from the luminal side of the media to the external side of the media, lamella #1 being the internal elastic lamina (see Figure 5 of the main article for illustration). (D) Mean of interlamellar thickness. (E) Number of elastic lamella disruptions. *general significant difference between *Eln*$^{+/+}$ and *Eln*$^{+/-}$ mice, independently of SEP treatment (three-way ANOVA, $p \leq 0.05$). In panel C, the symbol * next to the lamella number or mean thickness (M) indicates a significant difference between genotypes for the corresponding lamella or the mean only. Control animals were injected with the same volume of 0.9% NaCl (NaCl). Values are mean ± SEM. n=3-6 in each group.

Morphometric and quantitative analyses (CT-FIRE) of picrosirius red-stained collagen fibers in aorta cross-sections were then performed. Representative images are presented in Figure 7 of the main article. The thickness of the collagen fibers present in the entire wall was generally higher in females than males, and in *Eln*$^{+/-}$ than *Eln*$^{+/+}$ mice, independently of SEP treatment (Supplementary data S1.7A). When the analysis was restricted to the media, the collagen fibers were also found thicker in female than male animals, whereas no effect of genotype could be detected (Supplementary data S3A). While no effect of sex and genotype could be observed on the collagen fiber length in cross sections of the entire wall (Supplementary data S1.7B), collagen fibers were

generally found longer in females than males, and in *Eln*$^{+/+}$ than *Eln*$^{+/-}$ animals, when the analysis was restricted to the media (Supplementary data S3B). Collagen fiber straightness and angle were not changed by sex or genotype in the entire wall sections and when the analysis was restricted to the media (Supplementary data S1.7C,D and Supplementary data S3C,D). Finally, the collagen fiber number per aorta section was generally found lower in *Eln*$^{+/-}$ than *Eln*$^{+/+}$ mice, when the analysis was restricted to the media, not in the entire wall (Supplementary data S1.7E and Supplementary data S3E).

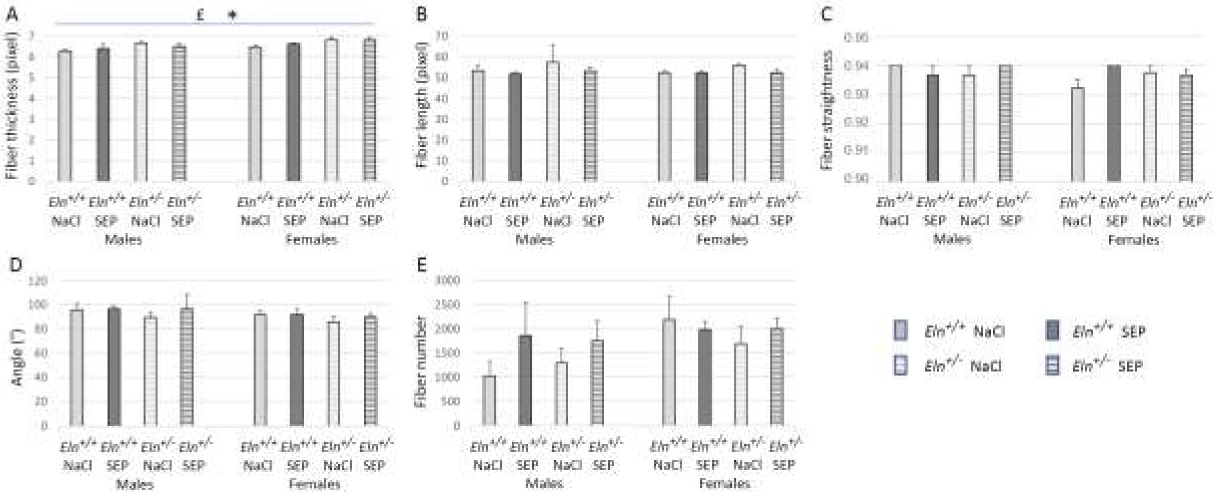

Supplementary data S1.7. CT-FIRE analysis of picrosirius red-stained cross-sections of the distal part of the aortic arch (entire aorta wall) illuminated with polarized light. The measurements were performed 3 days after NaCl (control) or SEP intravenous injection in the caudal vein of male and female mice of *Eln*$^{+/+}$ or *Eln*$^{+/-}$ genotypes. Individual properties and quantity of collagen fibers were compared between the studied groups: (A) fiber thickness, (B) length, (C) straightness, (D) angle and (E) number of fibers per aorta. *general significant difference between *Eln*$^{+/+}$ and *Eln*$^{+/-}$ mice, independently of treatment and sex (three-way ANOVA, p≤0.05). £general significant difference between male and female mice, independently of treatment and genotype (three-way ANOVA, p≤0.05). Control animals were injected with the same volume of 0.9% NaCl (NaCl). Values are mean ± SEM. n=3-6 in each group.

2.6. Aortic tissue mRNA levels

In order to have a wider view of the SEP impact on gene expressions in large elastic arteries and save animals, the gene transcripts were measured in thoracic and abdominal aorta segments by RT-qPCR. The mRNA levels were generally higher in *Eln*$^{+/-}$ than *Eln*$^{+/+}$ regarding all the genes, with the exception of *Lox*, independently of sex, treatment and vessel segment, and in males than females regarding *Loxl1*, *Fbln5* and *Col1a2* animals (Supplementary data S1.8). The mRNA levels were particularly elevated in male *Eln*$^{+/-}$ mice compared to their *Eln*$^{+/+}$ counterparts for *Fbln5* and *Loxl1*, independently of the vessel segment (Supplementary data S1.8E), and in the thoracic aorta only for *Eln* and *Col1a1* (Supplementary data S1.8A,F).

The mRNA levels were also generally found significantly higher in the abdominal aorta segment than in its thoracic counterpart for *Fbn1*, *Lox* and *Col1a2*, independently of animal sex, genotype and treatment

(Supplementary data S1.8B,C,G). Conversely, for *Col1a1*, mRNA levels were higher in the thoracic than in the abdominal aortic segment in *Eln*$^{+/-}$ male mice, while they were similar in abdominal or thoracic segments in *Eln*$^{+/+}$ mice of both sexes (Supplementary data S1.8F).

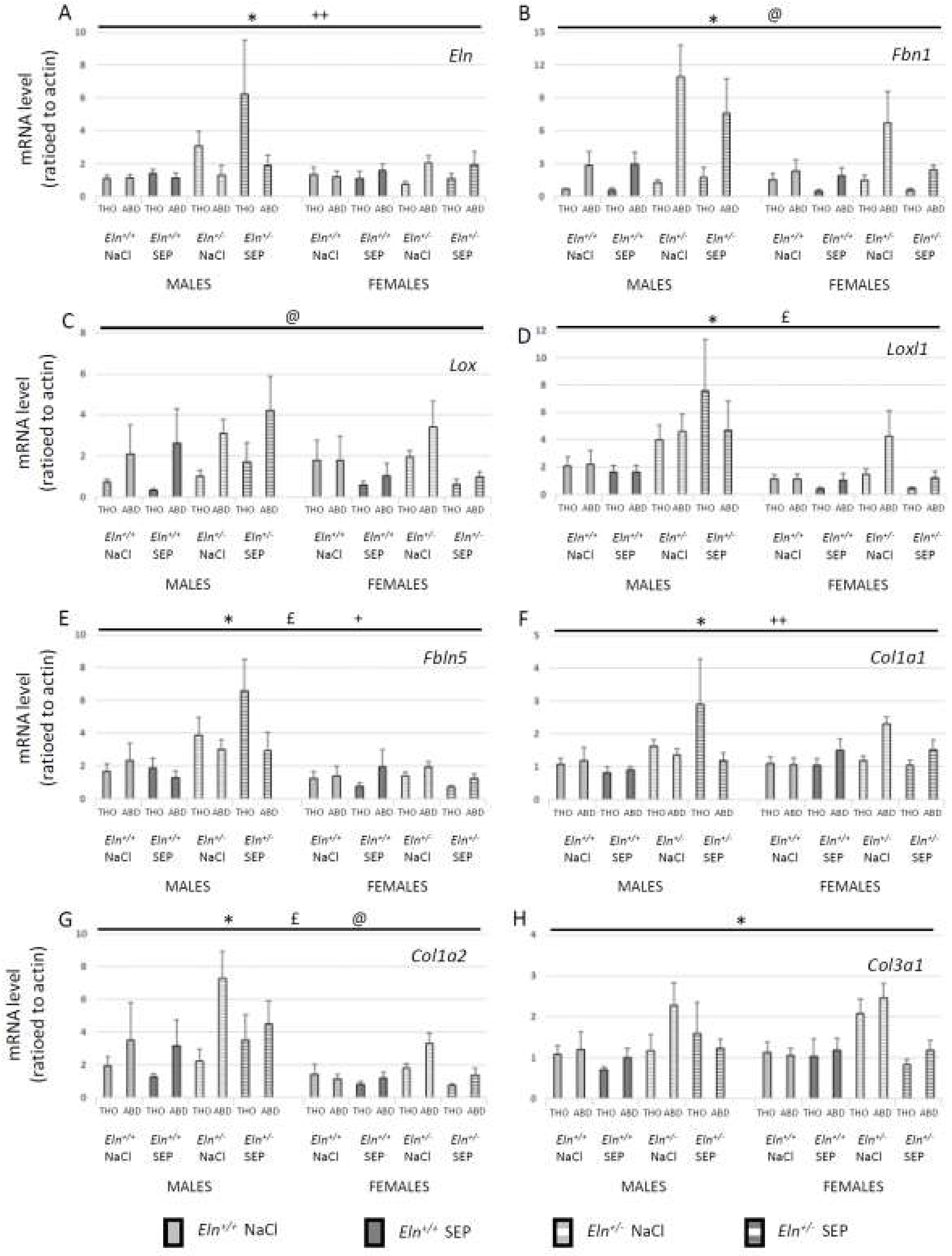

Supplementary data S1.8. Effect of SEP treatment on the levels of mRNAs expressed from the genes of the major components of aortic elastic and collagen fibers. The measurements were performed by RT-qPCR in descending

thoracic (THO) and abdominal (ABD) aorta segments, 3 days after SEP or NaCl (control) intravenous injection in the caudal vein of male and female mice of *Eln*$^{+/+}$ or *Eln*$^{+/-}$ genotype. (A) tropoelastin (*Eln*), (B) fibrillin-1 (*Fbn1*), (C) lysyl oxidase (*Lox*), (D) lysyl oxidase-like-1 (*Loxl1*), (E) fibulin-5 (*Fbln5*), (F) type I collagen alpha-1 (*Col1a1*), (G) type I collagen alpha-2 (*Col1a2*), (H) type III collagen alpha-1 (*Col3a1*). *general significant difference between *Eln*$^{+/+}$ and *Eln*$^{+/-}$ mice, independently of sex, SEP treatment and vessel segment (four-way ANOVA, p≤0.05), $^{£}$general significant difference between male and female animals, independently of genotype, SEP treatment and vessel segment (four-way ANOVA, p≤0.05), $^{@}$general significant difference between arterial segment type (THO vs. ABD), independently of sex, genotype and SEP treatment (four-way ANOVA, p≤0.05). $^{+}$significant interaction between sex and genotype (four-way ANOVA, p≤0.05), indicating that mRNA levels were higher in *Eln*$^{+/-}$ than *Eln*$^{+/+}$ mice in males only, independently of SEP treatment (post hoc Fisher's Least Significant Difference (LSD) tests, p≤0.05). $^{++}$significant interaction between sex, genotype and artery segment (four-way ANOVA, p≤0.05), indicating that the effect of genotype varies as a function of sex and artery segment (post hoc Fisher's Least Significant Difference (LSD) tests, p≤0.05). Control animals were injected with the same volume of 0.9% NaCl (NaCl). Values are mean ± SEM. n=5 in each group.

2.7. Aortic tissue protein levels

Western blotting of aorta extracts was performed for four major proteins involved in elastic fiber structure or synthesis: TE, FBN1, LOX and immature LOX (pre-LOX). Representative images are presented in Figure 10A of the main article. In the aorta wall, the levels of TE were generally found lower in *Eln*$^{+/-}$ than *Eln*$^{+/+}$ mice (Supplementary data S1.9A), while LOX levels were higher in *Eln*$^{+/-}$ than *Eln*$^{+/+}$ animals (Supplementary data S1.9C), independently of sex and treatment. In parallel, the levels of TE and LOX were generally lower in males than females (Supplementary data S1.9A,C), while the levels of pre-LOX were found higher in males than females (Supplementary data S1.9D), independently of genotype and treatment. Sex and genotype had no effect on FBN1 levels (Supplementary data S1.9B).

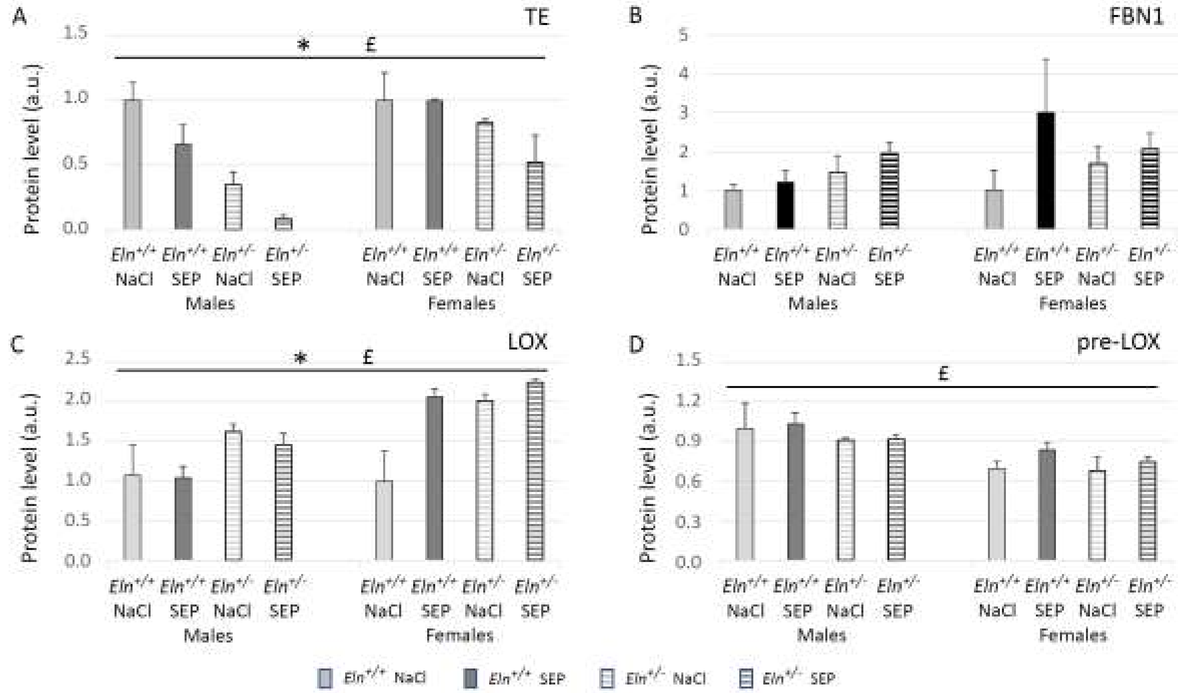

Supplementary data S1.9. Western blotting of descending thoracic aorta extracts from untreated or SEP-treated mice. Semi-quantitative analysis of the protein levels was performed by band densitometry for: A. tropoelastin

(TE), B. fibrillin-1 (FBN1), C. lysyl oxidase (LOX, 30kDa), D. immature lysyl oxidase (pre-LOX, 50kDa). *general significant difference between *Eln*$^{+/+}$ and *Eln*$^{+/-}$ mice, independently of sex and SEP treatment (three-way ANOVA, $p \leq 0.05$), £general significant difference between male and female mice, independently of genotype and SEP treatment (three-way ANOVA, $p \leq 0.05$). Control animals were injected with the same volume of 0.9% NaCl (NaCl). Values are mean ± SEM. n=5 in each group.

Supplementary data S2

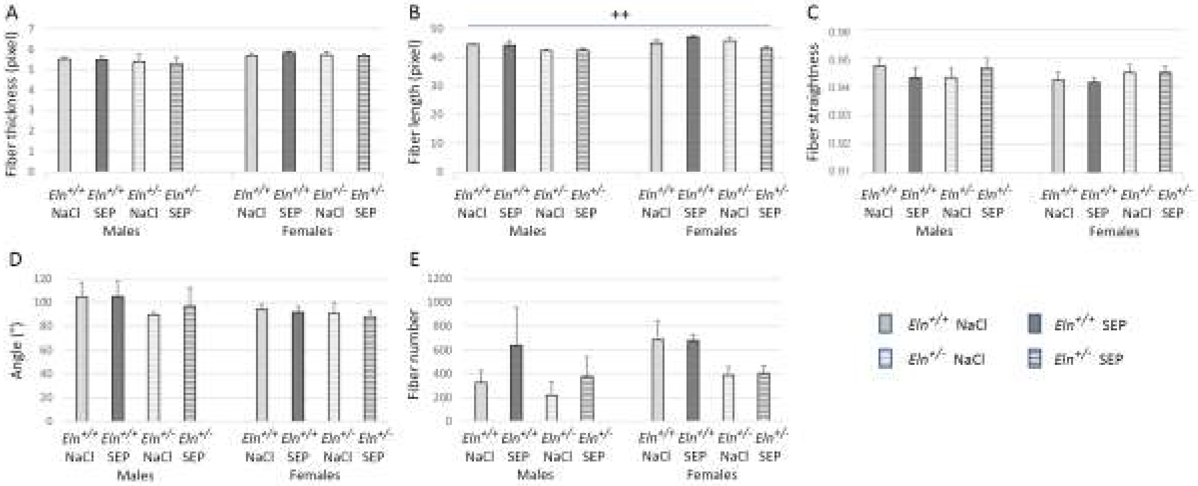

Supplementary data S2. CT-fire analysis of picrosirius red-stained cross-sections of the distal part of the aortic arch, restricted to the wall media, illuminated with polarized light: effect of SEP treatment. Individual properties and quantity of collagen fibers were compared between the studied groups: (A) fiber thickness, (B) length, (C) straightness, (D) angle and (E) number of fibers in the media per aorta. $^{++}$significant interaction between genotype, sex and SEP treatment (three-way ANOVA, $p \leq 0.05$), indicating that SEP treatment induced a collagen fiber length decrease in the aortic media of female $Eln^{+/-}$ and increase in female $Eln^{+/+}$ mice, while no change could be detected in male animals. Control animals were injected with the same volume of 0.9% NaCl (NaCl). n=3-6 in each group.

Supplementary data S3

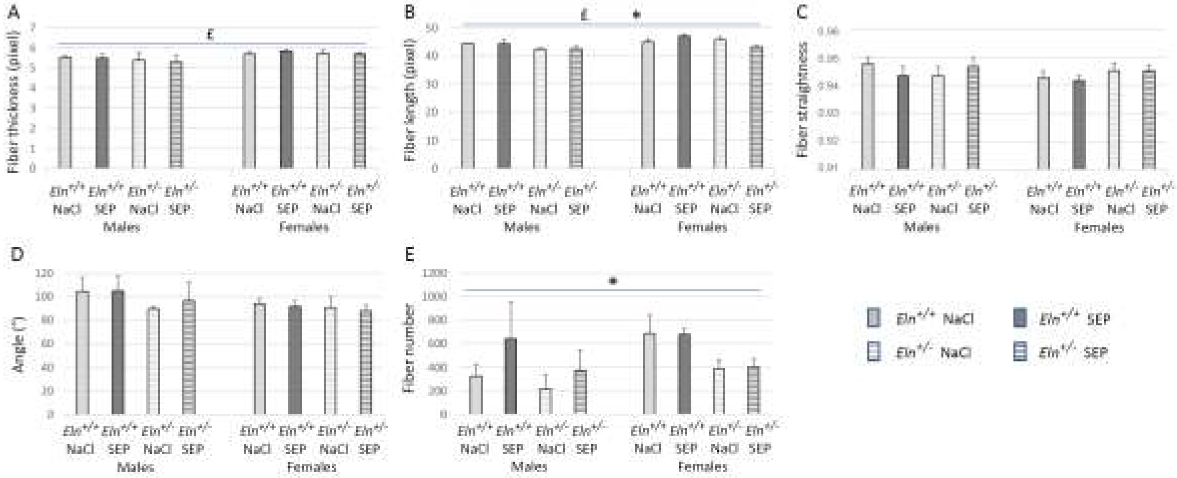

Supplementary data S3. CT-FIRE analysis of picrosirius red-stained cross-sections of the distal part of the aortic arch, restricted to the wall media, illuminated with polarized light: effects of genotype and sex. Individual properties and quantity of collagen fibers were compared between the studied groups: (A) fiber thickness, (B) length, (C) straightness, (D) angle and (E) number of fibers in the media per aorta. *general significant difference between *Eln*$^{+/+}$ and *Eln*$^{+/-}$ mice, independently of sex and SEP treatment (three-way ANOVA, $p \leq 0.05$). £general significant difference between male and female mice, independently of genotype and SEP treatment (three-way ANOVA, $p \leq 0.05$). Control animals were injected with the same volume of 0.9% NaCl (NaCl). n=3-6 in each group.

Supplementary data S4

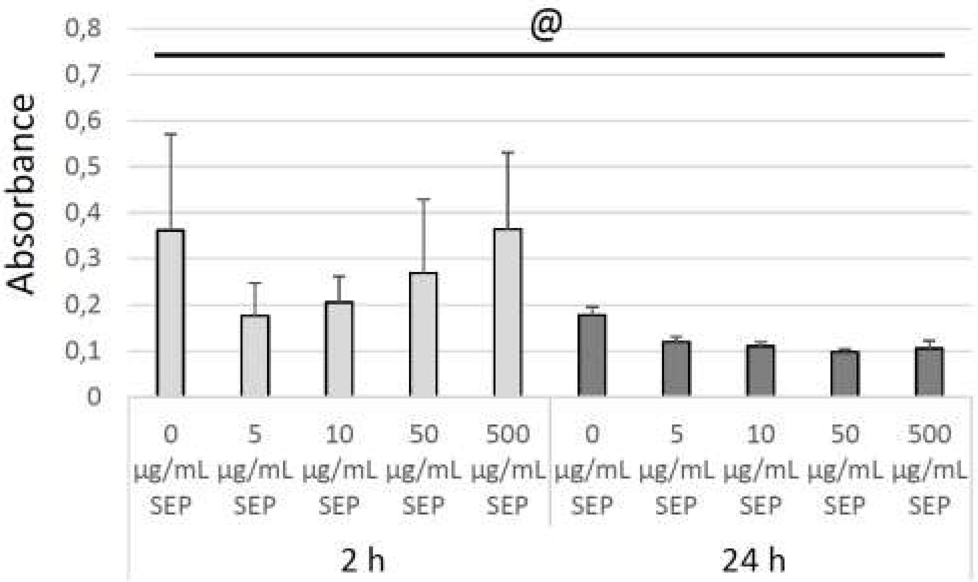

Supplementary data S4. ELISA detection of SEP-coated wells with anti-elastin antibodies. After 2h or 24h coating with SEP of wells from a 96-well plate, the same procedure as that described for detection of elastin in VSMC cultures was applied (see corresponding part of the materials and methods for details). The wells were coated with different concentrations of SEP (0, 5, 10, 50, 500 µg/mL) for 2h or 24h, then washed three times with 0.1% Tween containing PBS 1X. The primary antibodies to elastin (ab21610, Abcam), then the secondary antibodies coupled to HRP were applied to the wells, before chemical revelation and absorbance reading after 10 min. It was observed that the presence of SEP in the wells (5-500 µg/mL) did not significantly elevate the absorbance, compared to the wells with no SEP (0 µg/mL) (two-way ANOVA, $p=0.73$). Absorbance was even reduced in the presence of SEP after a 24h coating, compared to the absence of coating (one-way ANOVA, $p \leq 0.05$). It was concluded that the anti-elastin antibody did not recognize SEP. However, a general decrease in absorbance was detected between the two coating times, independently of the presence of SEP in the wells (@, two-way ANOVA, $p \leq 0.05$).
